# The long noncoding RNA *lnc-FAM164A1*-ACLY axis promotes pro-inflammatory responses in human primary macrophages: a systems approach

**DOI:** 10.3389/fimmu.2026.1776849

**Published:** 2026-05-01

**Authors:** Jian-Guo Wang, Shiori Ito, Daiki Hosokawa, Keishi Nihira, Julius L. Decano, Lang Ho Lee, Hideyuki Higashi, Arda Halu, Peter C. Mattson, Gabriela Amaral, Ana Luisa Abib, Alexander Mojcher, Andrew K. Mlynarchik, Mary Whelan, Daniel G. Anderson, Elena Aikawa, Sasha A. Singh, Shizuka Uchida, Masanori Aikawa

**Affiliations:** 1 The Center for Interdisciplinary Cardiovascular Sciences, Cardiovascular Division, Department of Medicine, Brigham and Women’s Hospital, Harvard Medical School, Boston, MA, United States; 2 Channing Division of Network Medicine, Department of Medicine, Brigham and Women’s Hospital, Harvard Medical School, Boston, MA, United States; 3 David H. Koch Institute for Integrative Cancer Research, Massachusetts Institute of Technology, Cambridge, MA, United States; 4 Department of Chemical Engineering, Massachusetts Institute of Technology, Cambridge, MA, United States; 5 Institute for Medical Engineering and Science, Massachusetts Institute of Technology, Cambridge, MA, United States; 6 Harvard-MIT Division of Health Science and Technology, Massachusetts Institute of Technology, Cambridge, MA, United States; 7 The Center for Excellence in Vascular Biology, Cardiovascular Division, Department of Medicine, Brigham and Women’s Hospital, Harvard Medical School, Boston, MA, United States; 8 Center for RNA Medicine, Department of Clinical Medicine, Aalborg University, Copenhagen, Denmark

**Keywords:** ACLY, inflammation, macrophages, NF-κB, noncoding RNA

## Abstract

**Introduction:**

Macrophages play a key role in inflammatory diseases. We aimed to identify long noncoding RNAs that regulate pro-inflammatory activation of human macrophages.

**Methods:**

We performed human lncRNA microarray analysis in LPS-stimulated primary human macrophages. We then carried out loss-of-function and gain-of-function experiments, luciferase reporter assays, RNA pulldown, RNA immunoprecipitation, and mouse endotoxemia studies, including humanized mouse models.

**Results:**

We identified 11 lncRNAs that were significantly increased by LPS. Among them, *lnc-FAM164A1* was selected for further study. Silencing of *lnc-FAM164A1* by antisense oligonucleotides or siRNA reduced the LPS-induced expression of pro-inflammatory cytokines, including CCL2, IL-6, and TNF-α. In contrast, enforced expression of *lnc FAM164A1* enhanced inflammatory responses in human and mouse macrophages. *lnc FAM164A1* also promoted NF-κB-related signaling. RNA pulldown and RNA immunoprecipitation identified ACLY as an *lnc-FAM164A1*-associated protein. ACLY silencing reduced the inflammatory effects induced by *lnc-FAM164A1*.

**Discussion:**

These findings support that human *lnc-FAM164A1* promotes pro-inflammatory activation of macrophages through its interaction with ACLY and NF-κB-related signaling.

## Introduction

Long noncoding RNAs (lncRNAs, >200nt), non-protein coding transcripts known as the “dark matter” of the genome, play unsuspected roles in numerous biological processes and human diseases, including embryonic development, tumor growth, metastasis, cardiometabolic disorders, and inflammation (1, 2). Based on the location to their neighboring protein-coding genes, lncRNAs are classified as antisense, intronic, divergent and intergenic lncRNAs (3–5). Although relatively less evolutionary conserved than protein coding genes (6), intergenic lncRNAs can regulate gene transcription in a tissue-specific, cell-type-specific, or disease-stage-dependent manner (7–9). LncRNAs can regulate biological functions by influencing the expression of nearby genes, organizing nuclear architecture or interacting with proteins and RNAs (10). LncRNAs regulate differentiation of immune cells, such as erythroid cells, T-lymphocytes, dendritic cells and monocytes/macrophages (11–13). Recent studies have advanced our understanding of lncRNAs in immunity, particularly their roles in macrophage biology, including regulation of polarization and inflammatory signaling. More research, however, is needed to fully uncover their diverse mechanisms (14, 15).

Macrophages are large phagocytes and a key player in innate immunity. Due to their ability to engulf pathogens, lipids, apoptotic cells, damaged tissue and to interplay with other immune cells, macrophages play an essential role in host defense and contribute to the pathogenesis of various inflammatory diseases. Macrophages are remarkably plastic and can switch their phenotype depending on the cellular stimulus in the tissue microenvironment. Alternatively, macrophage heterogeneity due to the balance of their subpopulations may shift in response to molecular cues, determining the inflammatory state of normal or pathological tissues (16–20). *In vitro*, cultured macrophages can be skewed into a pro-inflammatory phenotype using bacterial lipopolysaccharides (LPS) or interferon-gamma (IFN-γ) or an anti-inflammatory phenotype using interleukin (IL)-4 or IL-13. LPS recognizes toll-like receptor (TLR) 4 and activates macrophages. LPS-induced signaling triggers the phosphorylation and ubiquitin-dependent degradation of inhibitor of kappa B (IκB) proteins, resulting in the translocation of nuclear factor kappa B (NF-κB) dimers (e.g. p65/p50) from cytoplasm to nucleus and the production of pro-inflammatory mediators (e.g. IL-1β, IL-6, tumor necrosis factor alpha (TNF-α)).

Previous studies suggest certain lncRNAs regulate macrophage biology (21–24). Some lncRNAs, such as *lincRNA-Cox2 (*25) and *lincRNA-Eps (*26), have been studied in mouse macrophages, other lncRNAs, including *THRIL (*27), *RP5-833A20.1 (*28), *MacORIS (*29), *LUCAT1 (*30) and *CHROME (*31), have been investigated in human macrophages. The specific functions and underlying mechanisms of many lncRNAs in human macrophages, however, remain poorly understood, limiting their potential as therapeutic targets for inflammatory diseases (22, 32–34). Recent studies have highlighted *lnc-FAM164A1* (also referred to as *LINC02605*, *IL-7-AS*, or *RP11-79H23.3*), which is upregulated in THP-1 macrophages infected with *Campylobacter concisus*, suggesting potential involvement in antimicrobial or inflammatory pathways (35). In addition, *lnc-FAM164A1* enhances antiviral innate immune responses by enhancing IRF3 nuclear translocation, emphasizing its potential role in regulating cytokine-driven inflammation (36). *Lnc-FAM164A1* has also been associated with bladder cancer progression through *miR-107*-PTEN signaling (37). Despite these accumulating data, the molecular functions of *lnc-FAM164A1* in primary human macrophages, particularly during acute inflammatory activation, remain incompletely understood.

To identify intergenic lncRNAs that regulate macrophage activation in inflammatory diseases, we performed lncRNA microarray analysis of LPS-elicited human primary macrophages derived from PBMCs. Unbiased bioinformatic analysis detected 11 lncRNAs that significantly increased by LPS stimulation. We then selected intergenic *lncRNA-FAM164A1-1* (*lnc-FAM164A1*) for detailed functional and mechanistic investigations. Mechanistic studies revealed that human *lnc-FAM164A1* enhances the expression of pro-inflammatory molecules in part through NF-κB signaling in LPS-activated macrophages. We furthermore performed proteomics of RNA pulldown samples to identify *lnc-FAM164A1*’s interaction with ATP-citrate synthase (ACLY), which exerts pro-inflammatory responses in macrophages.

## Methods

### Reagents

Lymphocyte separation medium (LSM) was obtained from MP Biomedicals. Ultrapure LPS (tlrl-smlps, from *Salmonella*. Minnesota R595) was obtained from InvivoGen. The κB kinase (IKK) inhibitor (Bay11-7082) was obtained from Sigma-Aldrich. Antibodies used in Western blots were as follows: mouse monoclonal antibodies against β-actin (Novus Biologicals, NB600-501; 1:5000) and lamin A/C (Active motif, 39288; 1:500-4000), rabbit antibodies against p65 (Santa Cruz Biotechnology, sc-372; 1:200-1000), against IκB-α (Cell Signaling, 9242; 1:1000), against ACLY (Abcam, ab40793; 1: 5000 and Lifespan Biosciences, LS-B13453-50; 1:5000) and against hnRNPA1(ThermoFisher Scientific, PA5-19431; 1:500-1000). Antibodies used in RNA immunoprecipitation were as follows: rabbit monoclonal antibodies against ACLY (abcam, ab40793) and isotype control IgG (abcam, ab172730). pGL4.32[luc2P/NF-κB-RE/Hygro] Vector was purchased from Promega (E8491) and Dual-Glo Luciferase Assay System was from VWR (PAE2920). Lipofectamine^®^ 3000 Reagent (L3000015) was from Life Technologies. Pierce RIPA Buffer (PI-89900), Pierce IP Lysis Buffer (87787), Pierce™ RNA 3’ End Desthiobiotinylation Kit, Pierce™ Magnetic RNA-Protein Pull-Down Kit, and Halt Combined Protease and Phosphatase Inhibitor Cocktails (PI-78441) were from ThermoFisher Scientific.

### Cell cultures

#### THP-1 cells

The human monocytic leukemia cell line (THP-1) was purchased from ATCC (TIB-202) and cultured with Roswell Park Memorial Institute (RPMI) 1640 medium supplemented with 10% fetal bovine serum (FBS), penicillin/streptomycin in cell culture incubator at 37°C (5% CO2). THP-1 cells were plated in 12-well plates at 1.0 x 10^6^ per well and differentiated into macrophage-like cells by the addition of 100 ng/mL of phorbol myristate acetate (PMA) (Sigma-Aldrich P8139) for 48 hours.

#### Human primary macrophages derived from peripheral blood mononuclear cells

PBMCs were isolated from blood buffy coat (Research Blood Components, Brighton, MA) using lymphocyte separation medium (LSM) as described previously (64). PBMCs (5×10^6^ cells/well) were plated in 6-well culture plates and maintained in RPMI-1640 supplemented with 5% human serum and penicillin/streptomycin at 37°C (5% CO_2_) for 7–10 days before use. Whole blood of Rhesus monkey was purchased from Worldwide Primates, Inc.

#### Mouse peritoneal macrophages

Peritoneal macrophages were harvested 4 days after intraperitoneal injection of 2.5 mL of 4% Brewer thioglycolate medium (BD Diagnostic Systems, Sparks, MD) into C57BL/6J mice (8–10 weeks old, male, Jackson Laboratory) following euthanasia by CO_2_ inhalation using a gradual fill method with a displacement rate of 30% to 70% of the chamber volume per minute (65). 1x10^6^ cells were cultured on 12-well plates (Corning) with RPMI 1640 medium supplemented with 10% FBS and antibiotics (penicillin, streptomycin and amphotericin B). Non-adherent cells were washed with PBS after 1 hour culture.

#### Silencing of *lnc-FAM164A1* by antisense and siRNA oligonucleotides in human PBMC-derived macrophages

*Lnc-FAM164A1* RNA silencing was performed on human PBMC-derived macrophages using SilenceMag (BOCA Scientific, Boca Raton, FL) at final concentrations of 50–100 nM (66). 36–48 hours after transfection, macrophages were stimulated by 10 ng/mL LPS for 2–6 hours before collection of macrophages and cell culture medium. Anti-sense LNA™ long RNA GapmeR oligonucleotides target *lnc-FAM164A1* (Sequence 5’-GGTAAGAAATAGGTTG-3’) and nonspecific negative control oligonucleotides (Sequence 5’-AACACGTCTATACGC-3’) were designed by Exiqon A/S (Vedbaek, Denmark). The si-RNA oligonucleotides targeting *lnc-FAM164A1* and luciferase control siRNA oligos were designed by Axolabs (Kulmbach, Germany). Their sequences and chemical modifications are as follows (A, G, U, C: RNA nucleotide; a, g, u, c: 2’-O-methyl-nucleotide, s: Phosphorothioate, dT: deoxy-T residue): Luciferase control siRNA (XD-00194): Sense strand: 5’-cuuAcGcuGAGuAcuucGAdTsdT-3’; Antisense strand: 5’-UCGAAGuACUcAGCGuAAGdTsdT-3’. *Lnc-FAM164A1* specific siRNA oligo#5 (XD-05325): 5’-ucAccAuuAucuucAuAcudTsdT-3’; Antisense strand: 5’-AGuAUGAAGAuAAUGGUGAdTsdT-3’. *Lnc-FAM164A1* specific siRNA oligo#8 (XD-05328): 5’-uguGcuAAAAuAcuGAGAudTsdT-3’; Antisense strand: 5’-AUCUcAGuAUUUuAGcAcAdTsdT-3’.

### RNA extraction and real time-PCR

Total RNA was extracted using an Illustra RNAspin Mini kit (GE Healthcare, Piscataway, NJ) and the genomic DNA was removed by on-column DNase I digestion at room temperature for 15 minutes. cDNAs were synthesized using a high-capacity cDNA reverse transcription kit (Applied Biosystems, Carlsbad, CA). RT-PCR was performed on a 7900HT fast real-time PCR system (Applied Biosystems). Taqman probe and primers for *lnc-FAM164A1* were designed by Integrated DNA Technologies and listed as follows: Probe: 5’-/56-FAM/ATGG CCTCA/ZEN/GTTAGGACTGCTTGG/3IABkFQ/-3’; Primer1: 5’-GATGGGTGC TGGGAGTATAAG-3’; Primer2: 5’-CCGCTCTCTTTCTCTCTGTTAG-3’. Other Taqman probes for *IL-1β*, *IL-6*, *IL-10*, IL-7, *CCL2*, *TNF-α*, and *GAPDH* were purchased from Life Technologies. Relative expression of each gene was normalized by *GAPDH*.

### Global microarray screening

Human lncRNA microarray analysis was performed using RNA samples purified from PBMC-derived human primary macrophages. For microarray analysis, Agilent Array platform was employed. The data underwent unbiased bioinformatics analysis by the “limma” R package (67). For the heatmap visualization, expression values were standardized to Z-scores, calculated by subtracting the mean expression of a specific lncRNA across all samples from the individual expression value, and then dividing by the standard deviation.

### *In situ* hybridization using RNAscope

*Lnc-FAM164A1* RNA *in situ* was detected using RNAscope 2.5 Red Chromogenic assay (Advanced Cell Diagnostics, Inc., Catalog 322360) according to the manufacturer’s manual. The 20ZZ probe named Hs-lnc-FAM164A1 (Catalog 475881) was designed to detect the nucleotide sequences 412–1623 of *lnc-FAM164A1:1*. Briefly, human PBMC-derived macrophages were cultured on Millicell EZ slide (Millipore). After the stimulation with 10 ng/mL of LPS for certain hours, macrophage monolayers were fixed by 10% Neutral Buffered Formalin for 30 minutes at room temperature and then treated with hydrogen peroxide and Protease III before performing the RNAscope assay. After RNAscope staining, macrophages were stained with mouse anti-human CD68 (clone PG-M1, DAKO) at 4 degrees overnight and FITC-labelled goat anti-mouse IgG at room temperature for 1 hour. Slides were added mounting medium with DAPI (VECTASHIELD) (Vector Laboratory, Burlingame, CA, USA) before placing coverslips. Slides were examined using the confocal microscope A1 (Nikon) and the images were processed with Elements 3.20 software (Nikon).

### ELISA

Duoset ELISA kits (R&D systems, Minneapolis, MN) were used to detect the levels of cytokines (human and mouse CCL2, IL-6, and TNF-α) in culture medium from macrophages or mouse plasma.

### *Lnc-FAM164A1* RNA pulldown assays

*Lnc-FAM164A1* sense and antisense RNA were *in vitro* transcribed with T7 RNA polymerase (Invitrogen) and purified by RNA Clean & Concentrator™-5 (Zymo Research). RNA (10 µg) was labeled with biotin at 3’-terminus using Pierce™ RNA 3’ End Desthiobiotinylation Kit. The THP-1 differentiated macrophage lysates were prepared using Pierce IP Lysis Buffer supplemented with Anti-RNase and Protease/Phosphatase Inhibitor Cocktail. RNA pulldown was performed by using the Pierce™ Magnetic RNA-Protein Pull-Down Kit. Streptavidin Magnetic Beads were first prepared according to manufacturer’s instructions and then immediately subjected to the capture of biotin-labeled RNA in RNA capture buffer for 30 minutes at room temperature with agitation. The RNA-captured beads were incubated with 200 μg of cell lysates diluted in Protein-RNA Binding Buffer for 1 hour at 4°C with rotation. The RNA-binding protein complexes were washed three times with a washing buffer and then eluted by D-biotin Elution Buffer at 37°C for 30 minutes. The eluted protein complexes were denatured, reduced, and separated with 7.5% Mini-PROTEAN TGX™ Precast Protein Gels (Bio-Rad) with a running of 25% of gel length. After the Bio-Safe Coomassie Stain (Bio-Rad), the gel contained stained proteins were collected, washed, alkylated, and digested with trypsin at 37°C overnight for MS analysis. The eluted proteins were denatured and reduced in SDS-sample buffer (Boston Bioproducts) and used for Western blots.

### Mass spectrometry

The RNA pulldown samples were analyzed with the high resolution/accuracy Orbitrap Fusion Lumos mass spectrometer fronted with an EASY spray source and coupled to an Easy-nLC1000 HPLC pump (Thermo Scientific). The peptides were separated using a dual column set-up: an Acclaim PepMap RSLC C18 trap column, 75 µm×20 mm; and an EASY spray LC heated (45°C) column, 75 µm× 250 mm. The gradient was run at 300 nL/min from 5-21% solvent B (acetonitrile/0.1% formic acid) for 50 minutes, 21-30% Solvent B for 10 minutes, followed by five minutes of 95% solvent B. Solvent A was 0.1% formic acid. The instrument was set at 120 K resolution, and the top N precursor ions in 3 seconds cycle time (within a scan range of m/z 375-1500; isolation window 1.6 m/z) were subjected to collision induced dissociation (collision energy 30%) for peptide sequencing (MS/MS).

### Mass spectrometric data analysis

All MS/MS data were queried against the human UniProt database (downloaded on August 01, 2014; 88,994 entries) using the HT-SEQUEST search algorithm, via the Proteome Discoverer (PD) Package (version 2.1, Thermo Scientific). Methionine oxidation was set as a variable modification and carbamidomethylation of cysteine residues was set as a fixed modification. Peptides were filtered based on a 1% FDR (Percolator). Peptides assigned by Proteome Discoverer to a given protein group (Master Protein), and not present in any other protein group, were considered as unique and used for quantification. A minimum of two unique peptides were included. Quantification was done using the area-under-the-curve (AUC) of the extracted MS1 ion peak chromatograms of up to the top three most abundant peptides per protein (performed in PD2.1). Proteins that had more than two-fold change in sense versus antisense (S/AS) and sense versus beads(S/B) were considered.

### Western blot

Macrophage whole cell lysates were prepared using RIPA buffer containing protease inhibitor (Roche). Total protein was separated by 4–20% Mini-PROTEAN^®^ TGX™ Precast Gel and transferred using the iBlot Western blotting system (Life Technologies). Primary antibodies against IκB-α, p65, ACLY, LaminA/C and β-actin were used. Protein expression was detected using Pierce ECL Western Blotting substrate reagent (ThermoFisher Scientific) and ImageQuant LAS 4000 (GE Healthcare).

### Recombinant adenoviruses and transfection on macrophages

RNA was isolated from human PBMC-derived macrophages and converted into cDNA. *Lnc-FAM164A1* was amplified by PCR and then cloned into the pcDNA3.1 vector. *Lnc-FAM164A1* oligonucleotides were first amplified from pcDNA3.1-*lnc-FAM164A1*, sub-cloned into the pENTR11 entry vector, and then transferred to the pAd-CMV-V5 adenoviral vector through LR recombination using Gateway LR Clonase II Enzyme Mix to generate pAd/CMV*/lnc-FAM164A1* (Ad-*lnc-FAM164A1*) (Life Technologies, Grand Island, NY, USA). pAd/CMV/V5-GW/*lac*Z was used as control vector (68). Adenovirus was amplified by transfection of *Pac*I-digested vector in HEK293A cells with subsequent purification using Fast Trap Adenovirus Purification and Concentration (EMD Millipore) and dialysis in 10 mM Tris (pH 7.4) buffer with 10% (v/v) glycerol, and storage at −70°C. Viral titer was determined by plaque assay on 293 cells using Adeno-XTM rapid titer kit (Clontech, Mountain View, CA, USA) and expressed as infectious units (ifu). For the *in vitro* cultured primary macrophages and THP-1-differentiated cells, both Ad-lacZ and Ad-*lnc-FAM164A1* virus were added into the cells at 1000 ifu/macrophages and incubated for 48 hours. After a medium change, macrophages were stimulated with 10 ng/mL LPS for 0–3 hours before extraction of protein and mRNA for Western blot and RT-PCR, respectively.

### NF-κB-luciferase reporter assay

HEK293 cells were plated in 24-well plates (0.5x10^5^/well). On the next day, cells were co-transfected of pGL4.32 vector [luc2p/NF-κB-RE/Hygro] (Promega, E8491) with either pcDNA3.1 empty vector or pcDNA3.1-*lnc-FAM164A1* using Lipofectamine 3000. 24 hours later, the cells were stimulated by 10 ng/mL LPS for another 6 hours. Firefly luciferase activity was measured using Dual-Glo luciferase assay kit (Promega).

### Network analysis

To quantify the disease associations of the *lnc-FAM164A1* interacting candidate proteins, ACLY, FLG2 and SFPQ, the average shortest network distance between their modules and disease-related proteins were measured, where network distance is defined as the non-Euclidean distance measured in terms of the number of edges between two nodes. ACLY, FLG2 and SFPQ modules are defined as the subgraphs consisting of the respective candidate protein and its first neighbors, i.e., direct interaction partners, in the interactome. The average shortest distance D of a candidate module to disease genes is measured by calculating the shortest distance between each candidate module gene s and all genes t of a disease and then averaging over all candidate module genes s such that $\text{D}=\frac{1}{∥\text{S}∥}\sum_{\text{s}\in \text{S}}{\text{d}}_{\text{s}}$ and ${\text{d}}_{\text{s}}=\frac{1}{∥\text{T}∥}\sum_{\text{t}\in \text{T}}{\text{d}}_{\text{st}}$, where ${\text{d}}_{\text{st}}$ is the shortest distance between s and t and S and T are the sets of genes in the ACLY, FLG2 and SFPQ first neighbors modules and disease genes, respectively. To compare the average shortest distance value to random expectation, the average shortest distance of the same number of randomly selected genes to disease genes was calculated for N = 250 instances. To control for degree (i.e., the number of connections of a gene), the random selection was done in a degree-preserving manner where all genes were binned according to their degree and random genes were selected uniformly at random from their corresponding degree bin. Empirical p-values were calculated by ${\text{p}}_{\text{emp}}=\text{P}({\text{D}}_{\text{r}}<\text{D})$, where ${\text{D}}_{\text{r}}$ is the average shortest distance of the randomized instance. The interactome onto which the ACLY, FLG2 and SFPQ modules and disease genes were mapped consists of curated physical protein-protein interactions (PPIs) with experimental support, including binary interactions, protein complexes, enzyme-coupled reactions, signaling interactions, kinase-substrate pairs, regulatory interactions, and manually curated interactions from literature, as described previously (69). Disease genes were obtained from the DiseaseConnect (http://disease-connect.org) (70) (using entries with evidence from Genome-Wide Association Studies (GWAS) and Online Mendelian Inheritance in Man (OMIM) (http://www.omim.org/) and MalaCards (http://www.malacards.org/) (71) databases.

### Gain-of-function study by adenovirus in mouse model of endotoxemia

C57BL/6J mice (8–10 weeks old, male) were purchased from Jackson Laboratory. All animal experiments were approved by Institutional Animal Care and Use Committee at Beth Israel Deaconess Medical Center. 5x10^9^ ifu of Ad-lacZ or Ad-*lnc-FAM164A1* virus (0.2 mL) were intravenously injected into the mice through tail vein. After 3 days, mice were administered with 4 mg/kg of LPS into the peritoneum (72). All mice were then euthanized by CO_2_ inhalation using a gradual fill method with a displacement rate of 30% to 70% of the chamber volume per minute 3 hours after LPS challenge. Whole blood was collected from the inferior vena cava (IVC) into sodium citrate (final concentration: 0.38%). Mouse plasma was prepared by spinning at 4000 × g for 15 minutes. Tissue was collected or embedded in O.C.T. compound (Tissue-Tek) and then frozen in liquid nitrogen and stored at -80°C until further use.

### Conservation analysis

Conservation of the *lnc-FAM164A1* locus was assessed using the UCSC Genome Browser multi-species alignments (Multiz) and conservation tracks (phyloP/phastCons). Primate conservation was evaluated by the presence and extent of alignment in non-human primates (including rhesus macaque). To assess sequence-level conservation in mouse, we compared the human *lnc-FAM164A1* transcript sequence with the mouse lncRNA *GM16685* using BLASTN.

### Humanized mouse model of endotoxemia

Hu-CD34 NSG mice or Hu-CD34 NSG-SGM3 mice (4–5 months old, female) were purchased from Jackson Laboratory (#005557, #013062). NSG (NOD scid gamma) mouse strain was served as the host and transplanted with human CD34+ hematopoietic stem cells (HSCs). A small amount of blood was collected from Hu-CD34 NSG mice through intraorbital bleeding under isoflurane anesthesia delivered via a precision vaporizer (up to 5% for induction, followed by 1-3% for maintenance in oxygen). On the next day, these Hu-CD34 NSG mice were administrated intraperitoneally with 5 mg/kg of LPS and then euthanized by CO_2_ inhalation using a gradual fill method with a displacement rate of 30% to 70% of the chamber volume per minute 6 hours after LPS challenge. In the loss-of-function study, NSG-SGM3 mice were administered of 0.5 mg/kg lipid nanoparticle formulated siRNA against *lnc-FAM164A1* intravenously (twice in 5 days), followed by a bolus intraperitoneal injection of 5 mg/kg LPS. Whole blood was collected from the IVC into sodium citrate. Mouse plasma was prepared by spinning at 4000 × g for 15 minutes. Liver, spleen, and lung tissues were collected and stored at -80°C until further use. Total white blood cell (WBC) was prepared by using ACK (Ammonium-Chloride-Potassium) Lysing Buffer. Splenocytes were isolated as described previously (66). After incubation with anti-mouse CD16/CD32 mAb (BioLegend) to block the Fc receptor, cells were then stained with Alexa Fluor 488-conjugated human CD45, APC-Cy7-conjugated anti-mouse CD45 (BioLegend) in autoMACS running buffer containing bovine serum albumin, EDTA, PBS and 0.09% azide (Miltenyi Biotec) for 30 minutes. Stained cells were analyzed by the FACSAria2 (BD Bioscience) and Flowjo software.

### RNA immunoprecipitation

THP-1 cells and PBMC-derived macrophages were stimulated with 10 ng/mL LPS. For crosslinking, cell lysate was incubated in 1% formaldehyde in PBS for 10 minutes at room temperature. Cross-linking reaction was stopped by adding glycine to final concentration of 150 mM. After cross-linking, RNA immunoprecipitation was performed following the protocol of Magna RIP™ RNA-Binding Protein Immunoprecipitation Kit (Millipore Sigma). 5 μg/sample antibody was used for immunoprecipitation. RNA samples obtained in RIP were used for qPCR (RIP-qPCR).

### Statistical analysis

Data are shown as mean ± standard error (SE). “n” indicates the number of independent experiments or number of animals/samples. For comparisons between two groups, a one-tailed paired non-parametric test (Wilcoxon signed-rank test) was used. Differences between groups were analyzed using Dunn’s test to adjust for multiple comparisons. (GraphPad Prism 10, Prism Software Inc. La Jolla, CA). Results with *P* < 0.05, *P* < 0.01, or *P* < 0.001 were considered statistically significant.

## Results

### Global microarray screening and unbiased bioinformatic analysis identified *lnc-FAM164A1* in LPS-activated human primary macrophages

**Figure 1:**
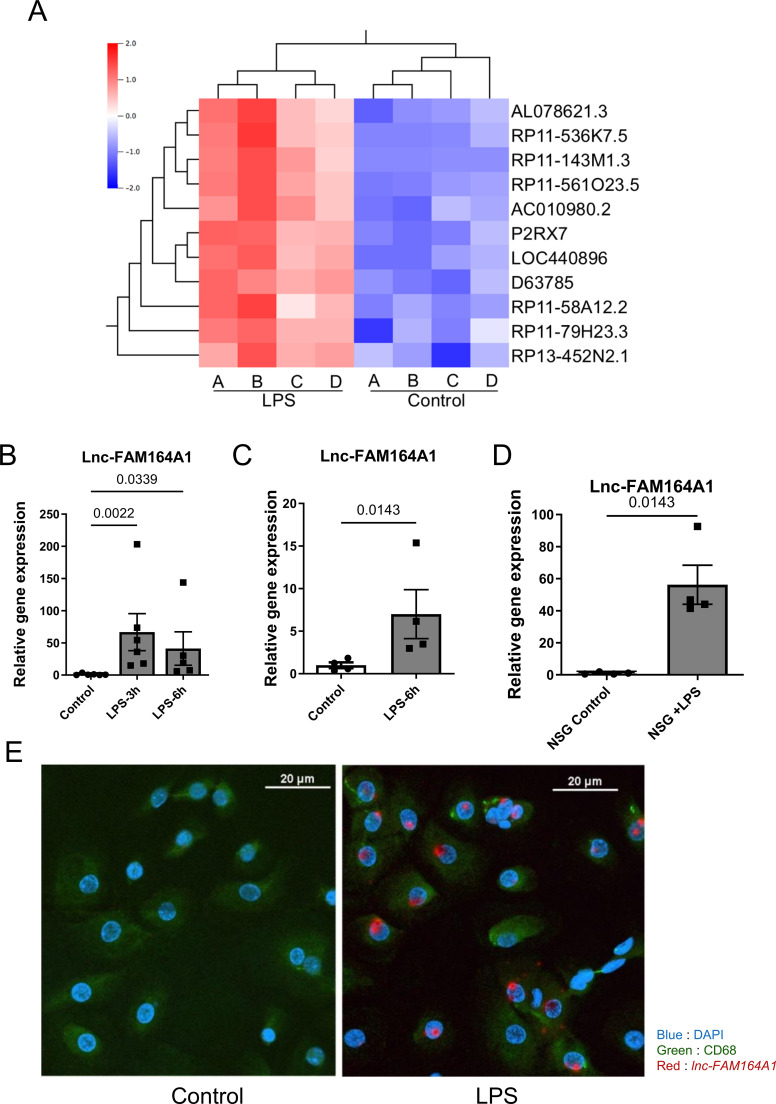
LPS treatment increases the expression of *lnc-FAM164A1* in human macrophages, PBMCs and white blood cells in humanized Hu-CD34 NSG mice. **(A)** Heatmap of hierarchical clustering analysis of the 11 lncRNAs (*RP11-79H23.3* or *lnc-FAM164A1*) showing a consistent response to LPS in 4 donors’ **(A–D)** PBMC-derived macrophages. The color scale represents the Z-score (defined in Methods), allowing for a clear visualization of relative expression patterns. **(B)** Human PBMC-derived macrophages stimulated with 10 ng/mL LPS for 3 hours or 6 hours (*P* < 0.05 LPS *vs* control; n= 5–6 donors; one LPS-treated sample was excluded due to insufficient RNA quality). **(C)** Human PBMCs isolated from citrated whole blood derived stimulated with 10 ng/mL LPS for 6 hours ex vivo (*P* < 0.05 LPS *vs* control; n= 4 donors). **(D)** Hu-CD34 NSG mice were stimulated with 5 mg/kg of LPS for 6 hours (*P* < 0.05, NSG control *vs* NSG +LPS, n = 4 mice per group). Relative fold change of the *lnc-FAM164A1* expression was determined by RT-PCR **(B–D)**. **(E)** Representative images of RNAscope *in situ* staining *of lnc-FAM164A1* on human PBMC-derived macrophages stimulated by LPS for 3 hours (Blue: DAPI; Red: *lnc-FAM164A1*; Green: human CD68; white bar scale: 20 μm).

To identify potential key regulators of macrophage activation, we explored early responsive non-coding transcripts in LPS-elicited human primary macrophages. We performed human lncRNA microarray analysis of RNA samples purified from PBMC-derived human primary macrophages, isolated from four different donors, 3 hours after LPS stimulation (**Supplementary Figure 1A** for the experimental design). LPS treatment produced statistically significant increases in the expression of 11 lncRNAs (FDR-adjusted p-value < 0.05 and fold-change ≥ 2) (**Figure 1A**, **Supplementary Figure S1B**), as also shown in the volcano plot (**Supplementary Figure 1C**). These 11 lncRNAs candidates included 7 intergenic, 2 antisense, 1 intro-sense overlapping and 1 exon-sense overlapping lncRNAs. We selected *lncRNA RP11-79H23.3* as an example to validate our systems approach. It is annotated as *lnc-FAM164A1* (*lnc-ZC2HC1A-1*, *LINC02605* or *IL-7-AS*) in LNCipedia (38), and is embedded within the locus of the non-coding RNA *MITA1*. Based on recent literature, we consider *lnc-FAM164A1* (*LINC02605*) and *MITA1* to be functionally related transcriptional isoforms originating from the same *IL-7-AS* locus (39). We focused on *lnc-FAM164A1* for further investigation because it exhibited one of the most robust and consistent increases after LPS stimulation across all four independent human primary macrophage donors. Furthermore, our selection was supported by the following factors: 1) it represents a specific, stable transcript of 2994 base pairs within this locus; and 2) its biological function in human macrophages remains largely unknown. The coding potential of *lnc-FAM164A1* was analyzed using the Coding Potential Assessment Tool (CPAT), the CPAT raw score 0.0059 suggests a noncoding RNA (< 0.364 coding probability cutoff) (38).

In human primary macrophages, LPS stimulation induced 67-fold and 41-fold increases in *lnc-FAM164A1* expression at 3 and 6 hours, respectively (RT PCR, **Figure 1B**). Pro-inflammatory cytokines IL-6 and TNF-α also increased the expression of *lnc-FAM164A1* in PBMC-derived macrophages (**Supplementary Figure 2A**). An approximate 7-fold increase in *lnc-FAM164A1* expression was also detected in human PBMCs isolated from LPS-stimulated whole blood *ex vivo* (**Figure 1C**). *Lnc-FAM164A1* was conserved among non-human primates (such as Rhesus monkey) as analyzed using the UCSC Genome Browser (**Supplementary Figure 2B**). Indeed, we found LPS induces a several-fold increase of *lnc-FAM164A1* in macrophages differentiated from PBMCs isolated from whole blood of Rhesus monkey (**Supplementary Figure 2C**).

Since *lnc-FAM164A1* has no homolog in mice, we established a humanized NSG mouse model of endotoxemia to further seek *in vivo* significance of this lncRNA. Three months after the transplantation with human CD34+ hematopoietic stem cells (HSCs), these humanized NSG mice contained 60-80% human CD45+ leukocytes in their blood (40). We found that LPS challenge increased the expression of human *lnc-FAM164A1* in white blood cells of humanized NSG mice (**Figure 1D**). We observed higher levels of human *lnc-FAM164A1* expression in the spleen than the lung of endotoxemic mice (**Supplementary Figure 2D**).

### LPS-induced *lnc-FAM164A1* localizes to the cytoplasm and the nuclei of macrophages

We then examined the localization of *lnc-FAM164A1* in macrophages. *In situ* hybridization using RNAscope (Advanced Cell Diagnostics) visualized the expression of *lnc-FAM164A1* RNA within macrophages. A low level of *lnc-FAM164A1* signal is located inside the nuclei of unstimulated macrophages, and LPS stimulation induced the expression of *lnc-FAM164A1* (**Figure 1E**).

*In situ* hybridization data show that LPS-induced *lnc-FAM164A1* is located in the cytoplasm and inside the nuclei. Indeed, by separating nuclear RNA and cytoplasmic RNA or subsequent relative quantification of *lnc-FAM164A1* expression using RT-PCR, we found that *lnc-FAM164A1* is distributed in both compartments (approximately 50% in the nucleus and 50% in the cytoplasm) at baseline. After LPS stimulation, its relative distribution shifts toward the cytoplasm (approximately 70-80% cytoplasmic). In contrast, the nuclear/cytoplasmic ratio of protein coding genes (*IL-1β* and *GAPDH*) are less than 5% (**Supplementary Figure 2E**), further confirming the subcellular localization of *lnc-FAM164A1*.

### LPS induces concomitant expression of *lnc-FAM164A1* with pro-inflammatory cytokines in macrophages

LPS triggers NF-κB signaling (41). Time-course experiments showed that *lnc-FAM164A1* has highest expression at 6 hours, with near baseline levels at 12 and 24 hours after the addition of LPS in human primary macrophages (**Supplementary Figure 3A**); similar to the expression profile of pro-inflammatory cytokines IL-1β, IL-6 and TNF-α, known targets of the NF-κB pathway (**Supplementary Figure 3A**). A specific inhibitor (Bay 11-7802) of NF-κB signaling blocked the transcription of *lnc-FAM164A1* and cytokines in a dose-dependent manner (3-30 μM) (**Supplementary Figure 3B**). These data indicates that *lnc-FAM164A1* is a target gene of NF-κB and may share similar or intertwined regulatory mechanisms with IL-1β, IL-6 and TNF-α in LPS-activated macrophages.

### Silencing of *lnc-FAM164A1* suppresses the expression of pro-inflammatory cytokines in LPS-activated human macrophages

**Figure 2:**
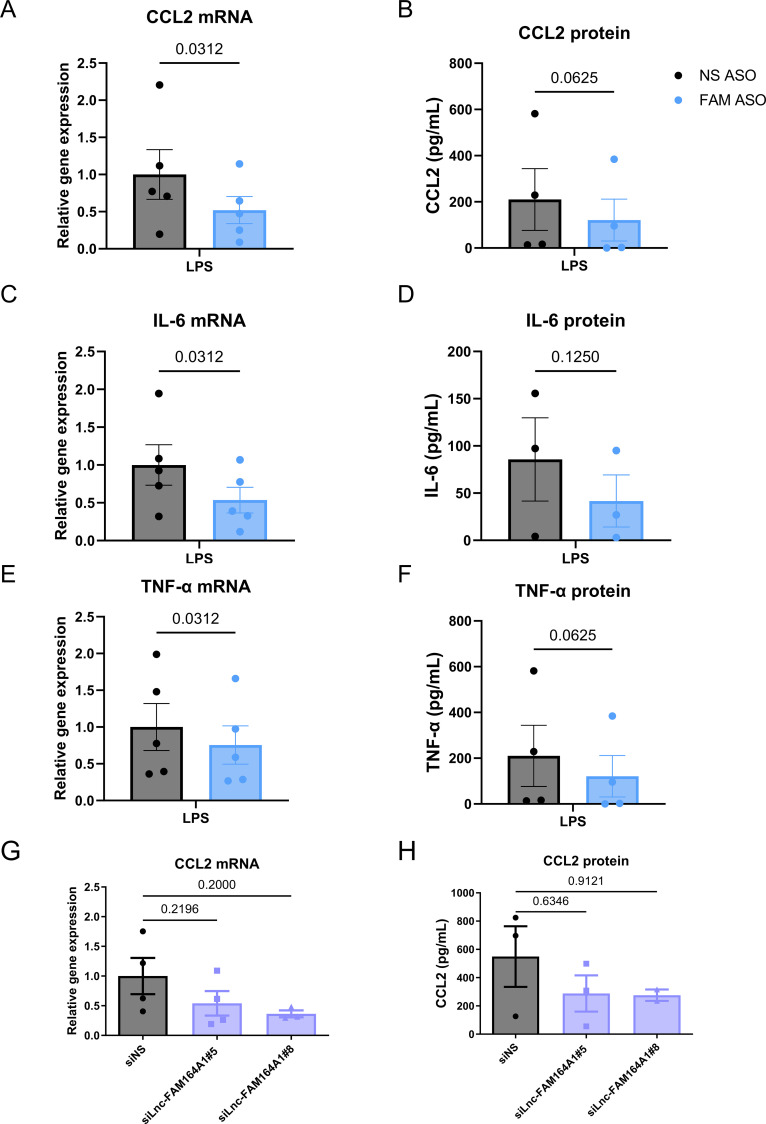
Silencing of *lnc-FAM164A1* reduces the expression of pro-inflammatory markers CCL2, IL-6, and TNF-α in LPS-stimulated human PBMC-derived macrophages. **(A–F)** Effects of FAM164A1 antisense oligonucleotide (FAM ASO) or non-specific antisense oligonucleotide (NS ASO) treatment on the expression of CCL2, IL-6, and TNF-α in LPS-stimulated human PBMC-derived macrophages. LPS treatment was followed by a 48-hour incubation with FAM ASO or NS ASO*. G and H*, Axolab siRNA oligonucleotides targeting *lnc-FAM164A1*(si*Lnc-FAM164A1*#5 and si*Lnc-FAM164A1*#8) or their nonspecific control oligonucleotides (siNS) treatment for 48 hours followed by 3–6 hours stimulation with LPS. mRNA expression **(A, C, E, G)** was determined by RT-PCR (*P* < 0.05 NS ASO-LPS *vs* FAM ASO-LPS; n = 3–5 donors). Protein **(B, D, F, H)** was determined by ELISA in culture media (*P* < 0.05 ASO-LPS *vs* FAM ASO-LPS; n = 2–4 donors).

We then determined whether *lnc-FAM164A1* plays a causal role in pro-inflammatory activation of macrophages by measuring cytokine and chemokine induction in LPS-activated human primary macrophages. To accomplish this, we performed loss-of-function experiments using anti-sense oligonucleotides (LNA™ long RNA GapmeR, Exiqon) that showed at least 50% silencing efficacy of *lnc-FAM164A1* in human macrophages (**Supplementary Figure 4A**). While *lnc-FAM164A1* silencing caused no changes in the expression of anti-inflammatory cytokine IL-10 and the neighboring gene *IL-7* in control and LPS-treated macrophages (**Supplementary Figures 4B, C**), the same treatment significantly suppressed the LPS-induced mRNA expression of the pro-inflammatory chemokine C-C motif chemokine ligand 2 (*CCL2*) and cytokines *IL-6* and *TNF-α*, and protein release of these cytokines showed a downward trend (**Figures 2A–F**). Inspired by cytoplasmic location of *lnc-FAM164A1*, we also designed 10 different siRNA oligonucleotides targeting *lnc-FAM164A1* and determined their silencing efficacy in human macrophages (Axolabs, Kulmbach, Germany). Oligonucleotides #5 and #8 showed the highest potency of silencing amongst the 10 oligonucleotides (**Supplementary Figure 4D**). siRNA silencing of *lnc-FAM164A1* with these oligonucleotides also resulted in decreased expression of CCL2 (**Figures 2G, H**) and IL-6 (**Supplementary Figures 4E, F**) at mRNA and protein levels in LPS-stimulated human macrophages. No significant change of *TNF-α* mRNA was detected (**Supplementary Figure 4G**). Due to the genomic overlap between *lnc-FAM164A1* (*LINC02605*) and *MITA1*, the ASOs and siRNAs used in this study likely targeted shared exonic regions, potentially reducing the expression of both isoforms. Therefore, to validate the functional of the *lnc-FAM164A1* sequence itself, we next performed gain-of-function experiments.

### Enforced expression of *lnc-FAM164A1* enhances LPS-induced TNF-α expression in macrophages

**Figure 3:**
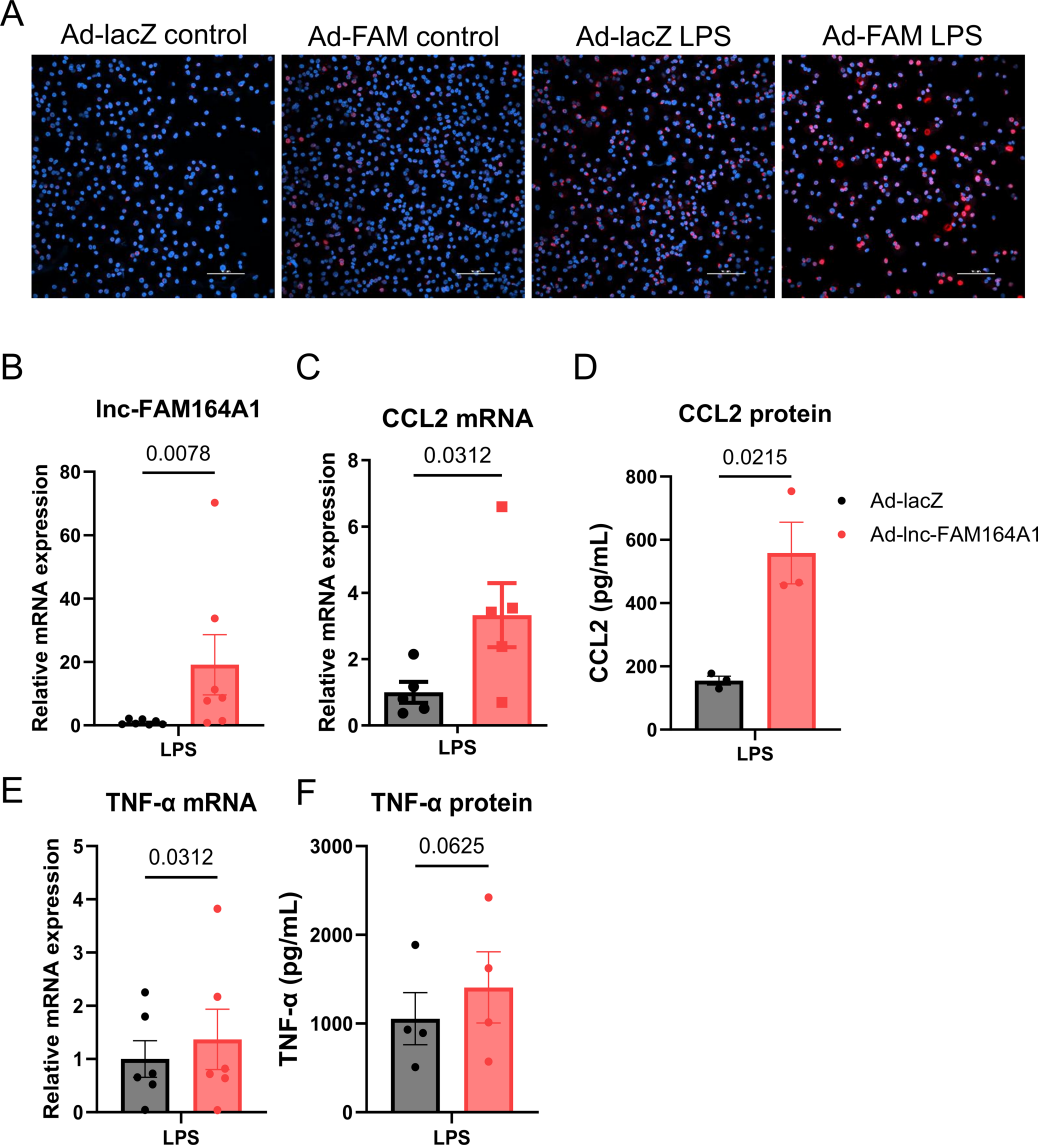
Enforced expression of *lnc-FAM164A1* enhances the expression of pro-inflammatory TNF-α and CCL2 induced by LPS on human PBMC-derived macrophages. **(A)** Human PBMC-derived macrophages infected with LacZ control adenovirus (Ad-lacZ) or *lnc-FAM164A1* expressing adenovirus (Ad-*lnc-FAM164A1*) for 48 hours and then stimulated with 10 ng/mL LPS for 2 hours. Representative images of RNAscope *in situ* staining of *lnc-FAM164A1* on adenovirus infected human PBMC-derived macrophages stimulated with or without LPS for 2 hours (Blue: DAPI; Red: *lnc-FAM164A1*; magnification 20x; white bar scale: 100 μm). **(B)** Expression of *lnc-FAM164A1* RNA; **(C)** *CCL2 mRNA;* **(D)** CCL2 protein; **(E)** *TNF-α* mRNA; and **(F)** *TNF-α* protein when PBMC-derived macrophages were treated with Ad-LacZ or Ad-*lnc-FAM164A1* in control versus LPS conditions. mRNA expression was determined by RT-PCR and protein by ELISA in culture medium (*P<0.01 or P<0.05* Ad-LacZ *vs* Ad-*lnc-FAM164A1*; n = 3–7 donors).

To further determine the causal role of *lnc-FAM164A1* in pro-inflammatory activation of macrophages, we performed gain-of-function experiments using adenovirus expressing *lnc-FAM164A1* (Ad-*lnc-FAM164A1*) or lacZ control in the human macrophage-like cell line THP-1 and human PBMC-derived macrophages. As compared with unstimulated THP-1 cells, Ad-*lnc-FAM164A1* produced a 20-fold induction of *lnc-FAM164A1* as compared with LacZ Control (**Supplementary Figure 5A**), which had no significant effects on the basal levels of TNF-α (**Supplementary Figures 5B, C**) and CCL2 (**Supplementary Figures 5D, E**). LPS treatment enhanced *lnc-FAM164A1* expression in THP-1 cells infected by Ad-*lnc-FAM164A1* (**Supplementary Figure 5A**), which concomitantly associated with significant increases in *TNF-α* and *CCL2* mRNA expression, and TNF-α protein release in the culture media (**Supplementary Figures 5B–E**). We observed no significant changes in the expression of IL-1β and decreased expression of IL-6 protein release in culture medium (**Supplementary Figures 5F–I**). Similarly, in human PBMC-derived macrophages, RNAscope *in situ* hybridization visually confirmed the successful enforced expression of *lnc-FAM164A1* (**Figure 3A**). Enforced expression of *lnc-FAM164A1* (**Figure 3B**) enhanced LPS-triggered CCL2 expression (**Figures 3C, D**). Enforced expression of *lnc-FAM164A1* in LPS-stimulated macrophages did not result in a significant change in TNF-α mRNA and protein levels compared to Ad-lacZ control (**Figures 3E, F**). No significant changes in the expression of IL-6 and IL-1β were observed (**Supplementary Figures 5J–L**).

### *Lnc-FAM164A1* enhances NF-κB signaling in LPS-activated macrophages

**Figure 4:**
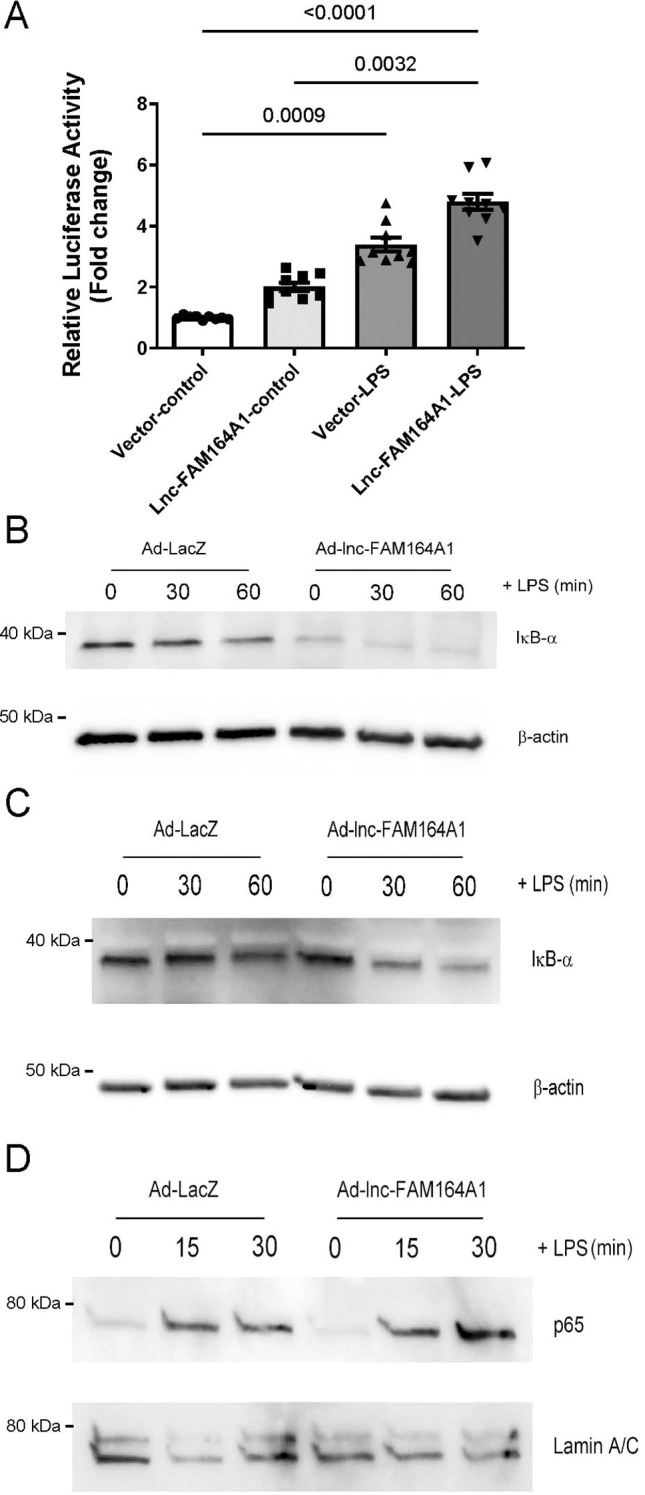
Enforced expression of *lnc-FAM164A1* promotes the activation of NF-κB signaling pathway in macrophages. **(A)** Enforced expression of *lnc-FAM164A1* enhances the activation of NF-κB promoter assessed by NF-κB-luciferase reporter assay in HEK293 cells (*P* < 0.05 Vector vs *Lnc-FAM164A1*, n = 9 cell culture replicates). **(B)** Enforced expression of *lnc-FAM164A1* by adenovirus enhances the degradation of IκB-α in THP-1 macrophages; and **(C)** in human PBMC-derived macrophages up to 60 minutes of LPS induction. **(D)** Enforced expression of *lnc-FAM164A1* also increased the nuclear accumulation of p65 in human PBMC-derived macrophages up to 60 minutes of LPS induction. IκB-α and p65 protein in nuclear extracts were detected by Western blots. β-actin and laminA/C were used as loading controls.

The NF-κB pathway plays a pivotal role in the transcriptional activation of pro-inflammatory factors, such as IL-1β, IL-6 and TNF-α in LPS-stimulated macrophages. Ligation of TLR4 with bacterial LPS triggers the signal transduction to induce the phosphorylation and the degradation of IκB proteins, followed by NF-κB dimers translocating from cytoplasm to nucleus (41). Our loss-of-function and gain-of-function data indicate that *lnc-FAM164A1* may promote TNF-α expression through its interaction with the NF-κB pathway. To test our hypothesis, we used a luciferase reporter assay in HEK293 cells co-transfected with either empty vector or *lnc-FAM164A1* expression vector combined with pGL4.32[luc2P/NF-κB-RE/Hygro] vector that contains five copies of NF-κB response element (NF-κB-RE) driving the transcription of luciferase reporter gene (luc2P). Overexpression of *lnc-FAM164A1* increased the luciferase activity in unstimulated or LPS-activated cells (**Figure 4A**). We then performed Western blot analysis to detect the protein levels of IκB-α in whole-cell lysates and p65 transcription factor in the nuclear extracts. Expression of *lnc-FAM164A1* by adenovirus infection induced rapid IκB-α degradation induced by LPS on both THP-1 cells (**Figure 4B**) and human PBMC-derived macrophages (**Figure 4C**). Further, enforced expression of *lnc-FAM164A1* increased the p65 translocation and accumulation in the nucleus 30 minutes after LPS stimulation in human PBMC-derived macrophages (**Figure 4D**). These results indicate that *lnc-FAM164A1* may regulate the expression of pro-inflammatory molecules through NF-κB signaling.

### Enforced expression of *lnc-FAM164A1* enhances TNF-α expression in a mouse model of endotoxemia *in vivo*

**Figure 5:**
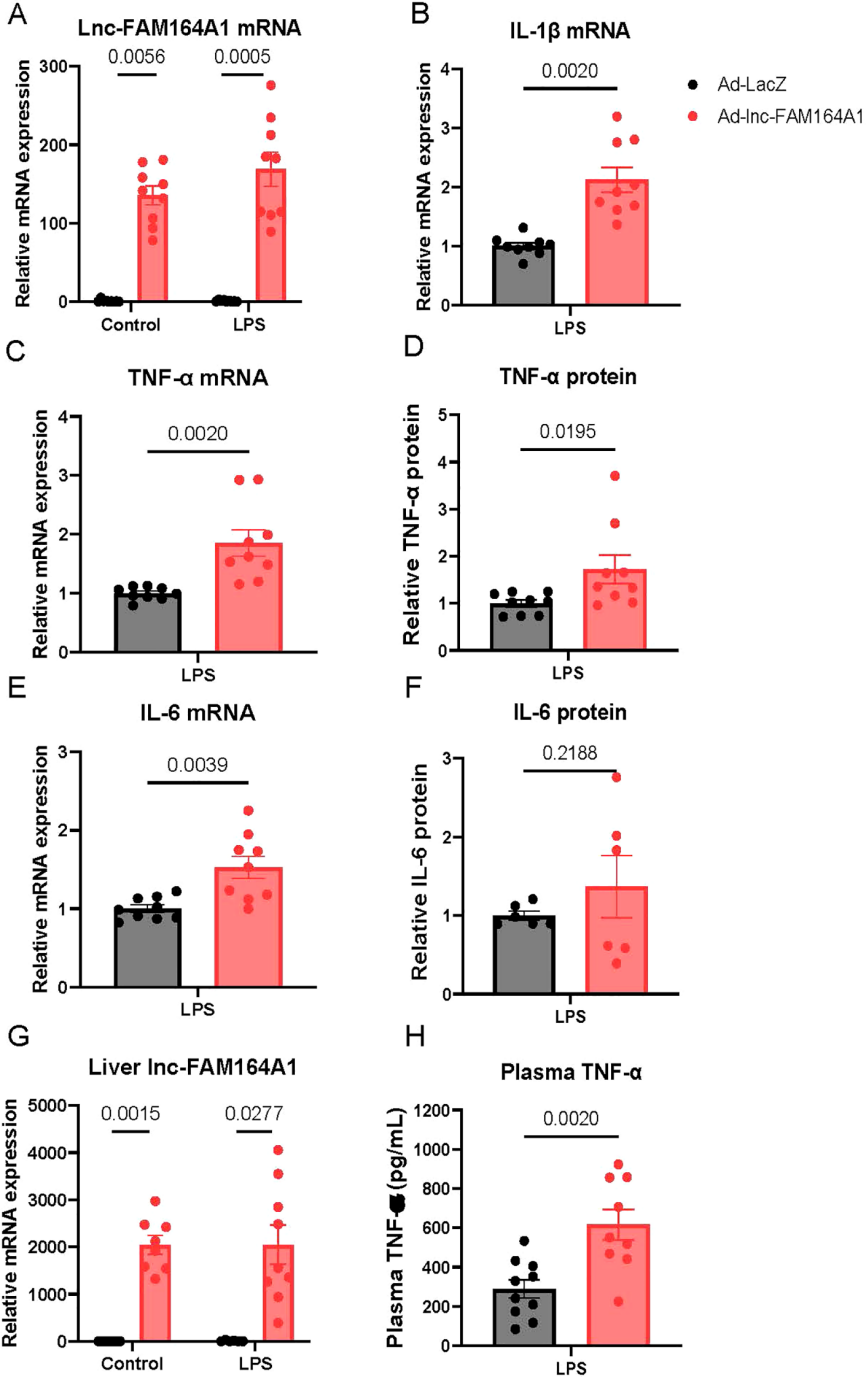
Enforced expression of *lnc-FAM164A1* enhances the induction of pro-inflammatory cytokines induced by LPS on mouse peritoneal macrophages. **(A–F)** Mice infected with LacZ control adenovirus (Ad-lacZ) or *lnc-FAM164A1* expressing adenovirus (Ad-*lnc-FAM164A1*) for 48 hours and then stimulated with 10 ng/mL LPS for 3 hours. mRNA expression **(A, C, E)** was determined by RT-PCR and protein levels by ELISA in culture medium **(B, D, F)** (*P* < 0.05 Ad-LacZ vs Ad-*lnc-FAM164A1*; n = 6–9 mice per treatment group). **(G, H)** Enforced expression of *lnc-FAM164A1* enhances the induction of plasma TNF-α induced by LPS in mouse model of endotoxemia. C57BL/6J mice were infected with LacZ control adenovirus (Ad-lacZ) or *lnc-FAM164A1* expressing adenovirus (Ad-*lnc-FAM164A1*) for 3 days and then stimulated with LPS for 3 hours. **(G)** Expression of *lnc-FAM164A1* RNA in the liver tissue was determined by RT-PCR. *H*, Plasma levels of murine TNF-α protein was measured by ELISA. (*P* < 0.05 Ad-LacZ+LPS *vs* Ad-*lnc-FAM164A1*+LPS; n = 7–10 mice per group).

In mouse, direct sequence comparison between human *lnc-FAM164A1* and the mouse *IL-7-AS* showed only short local alignments with low query coverage (~8%), which does not support sequence-level conservation of the full transcript. It is plausible the interacting protein(s) of *lnc-FAM164A1* are conserved between human and mouse, which provides us the rationale to perform gain-of-function studies in peritoneal macrophages isolated from wild-type C57BL6 mice. Ad-*lnc-FAM164A1* induced high levels of *lnc-FAM164A1* expression in mouse peritoneal macrophages (**Figure 5A**). Consistent with the results found in human macrophages (**Figure 3**), enforced expression of human *lnc-FAM164A1* enhanced the expression of *IL-1β*, *TNF-α*, and *IL-6* mRNAs (**Figures 5B, C, E**) and TNF-α protein (**Figure 5D**) in LPS-elicited peritoneal macrophages without affecting the basal levels of these molecules in unstimulated cells. In contrast, no changes of *CCL2* mRNA (**Supplementary Figure 6A**) and IL-6 protein (**Figure 5F**) were observed.

To determine whether *lnc-FAM164A1* regulates pro-inflammatory gene expression *in vivo*, we conducted gain-of-function experiments using a well-established mouse model of endotoxemia (42). Three days after the administration of adenovirus, enforced expression of *lnc-FAM164A1* was detected in the liver by RT-PCR (**Figure 5G**). We found that adenovirus infection alone had no effects on plasma levels of the pro-inflammatory cytokines TNF-α and IL-6 (ELISA, **Figure 5H**, **Supplementary Figure S6B**). However, a significantly enhanced level of TNF-α was detected in the plasma of endotoxemic mice infected with Ad-*lnc-FAM164A1* after 3-hour stimulation with LPS (**Figure 5H**). No significant change of plasma IL-6 protein was detected by ELISA (**Supplementary Figure 6B**). Endotoxemic mice infected with Ad-*lnc-FAM164A1* have significantly higher mRNA levels of cytokines in the liver (**Supplementary Figures 6C–E**).

### siRNA silencing of *lnc-FAM164A1* in human monocyte/macrophages decreases human CCL2 and IL-6 expression in a humanized mouse model of endotoxemia

**Figure 6:**
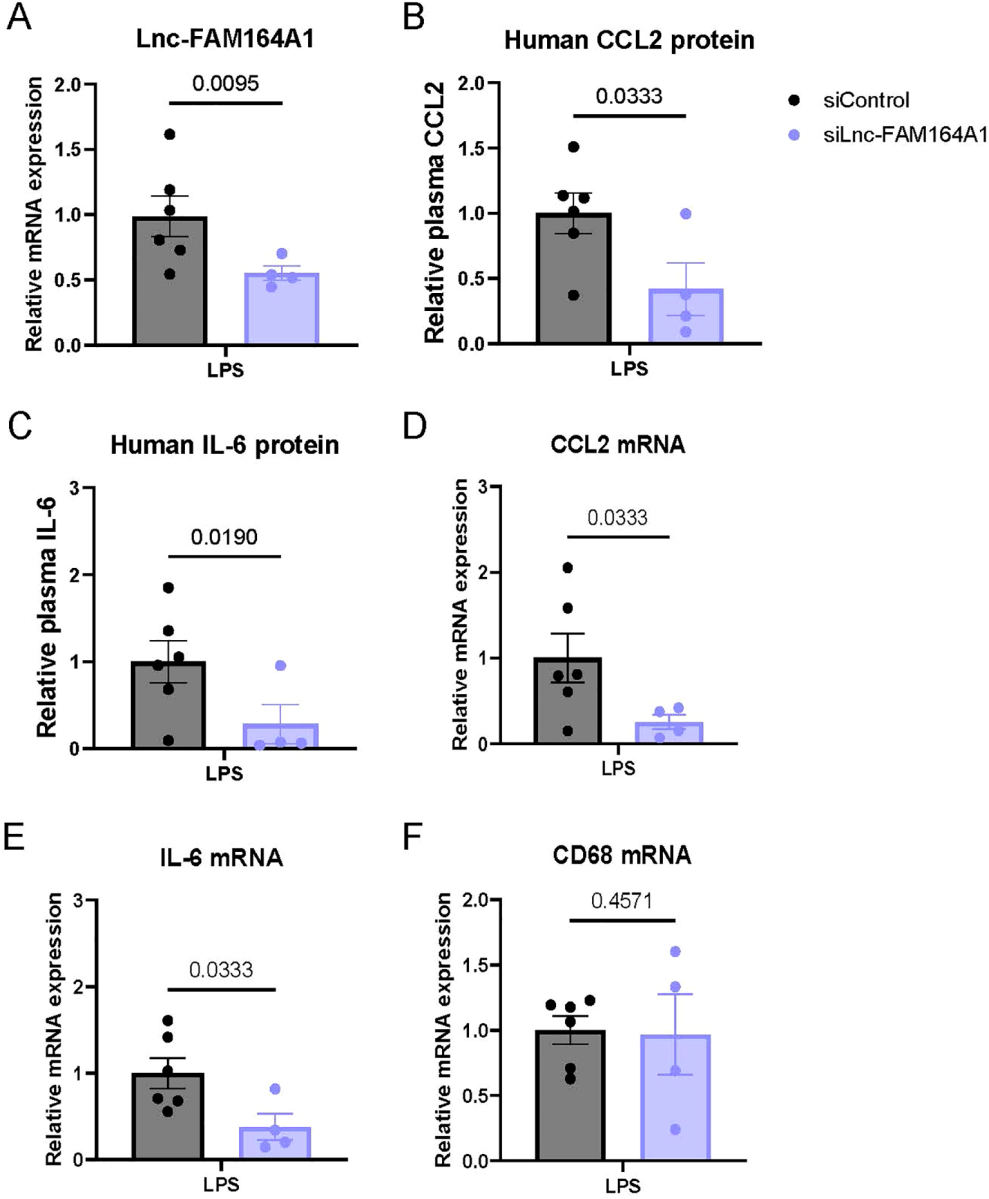
Silencing *lnc-FAM164A1* in monocytes/macrophages reduces human CCL2 and IL-6 expression in a humanized mouse model of endotoxemia. **(A)** Expression of *lnc-FAM164A1* RNA in monocytes derived from Hu-CD34 NSG-SGM3 mice administrated with lipid nanoparticle formulated siRNA control oligos or si-lnc-FAM oligos, followed by LPS challenge for 3 hours. **(B, C)**. Plasma levels of human CCL2 and IL-6 protein as detected by ELISA. **(D-F)**, *CCL2*, *IL-6* and *CD68* mRNA in peritoneal cells isolated from the endotoxemic mice as determined by RT-PCR. *P* < 0.05, si-control +LPS *vs* si-*lnc-FAM* +LPS (n = 4–6 mice per group).

In order to determine the *in vivo* significance of *lnc-FAM164A1* in human monocytes/macrophages, we explored the loss-of-function study in recently developed humanized NSG-SGM3 mice, which bear 30-80% human CD45+ WBCs and 2-12% of monocytes in the blood, spleen and bone marrow after the transplantation with human CD34+ HSCs (**Supplementary Figure 7**) (40). *lnc-FAM164A1* siRNA oligos or control siRNA oligos encapsulated in monocyte/macrophage-targeted lipid nanoparticles C12–200 were administrated to humanized NSG-SGM3 mice. We found a 30% decrease of *lnc-FAM164A1* expression in purified human monocytes from lnc-FAM siRNA treated mice compared with control mice (**Figure 6A**). *Lnc-FAM164A1* siRNA-treated mice have significantly lower levels of human CCL2 and IL-6 protein in plasma (**Figures 6B, C**), and decreased expression of human *CCL2* and *IL-6* mRNA in peritoneal cells (**Figures 6D, E**). No change of human *CD68* mRNA was detected (**Figure 6F**) in peritoneal cells, which suggests a similar number of human macrophages accumulated in LPS-stimulated peritonitis.

### RNA pulldown combined with mass spectrometry identified three proteins associated with *lnc-FAM164A1*

**Figure 7:**
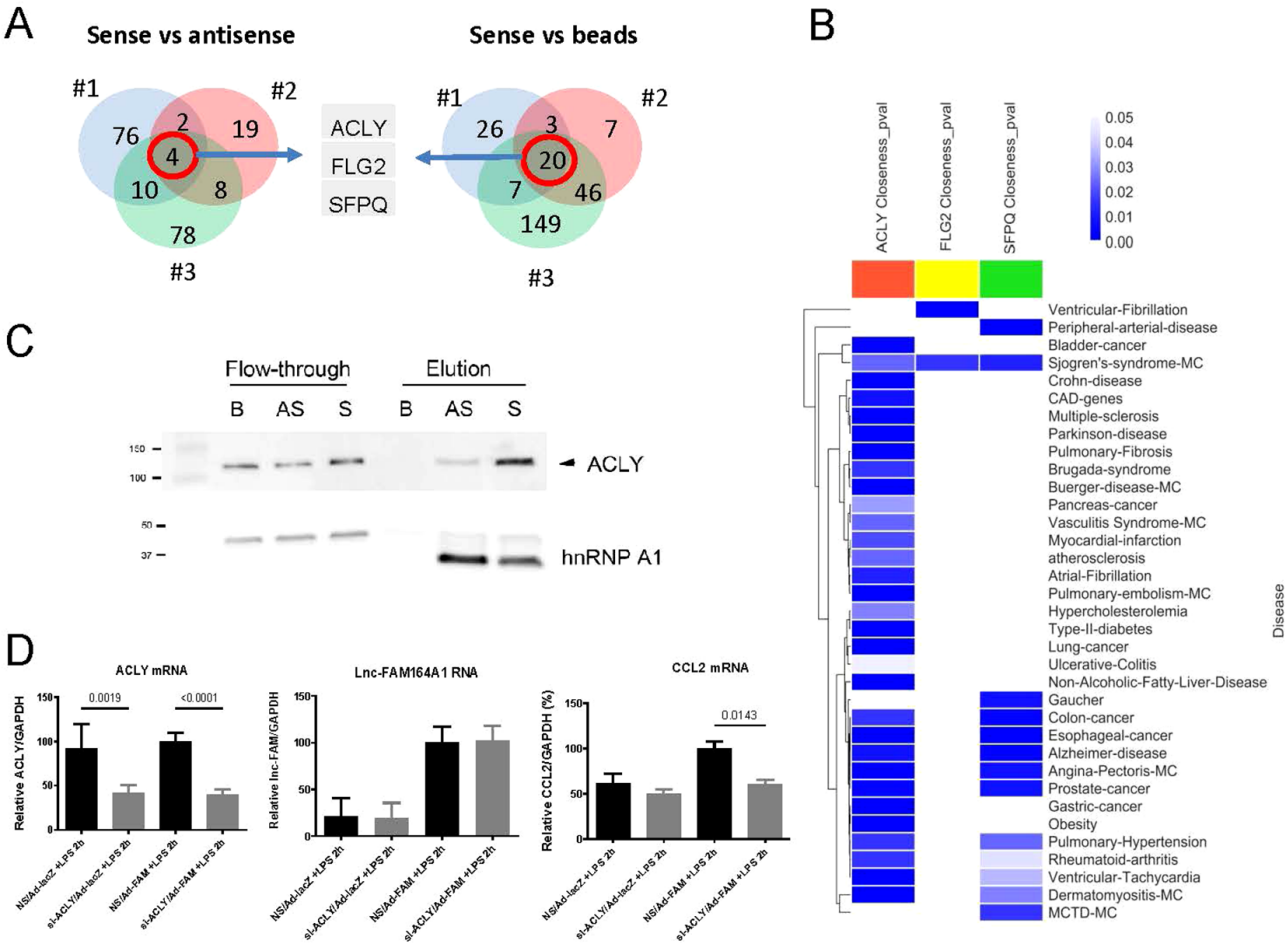
RNA pulldown of *lnc-FAM164A1* identifies three interacting proteins. **(A)** Venn diagram comparing the number of proteins identified in three *lnc-FAM164A1* RNA pulldown experiments (#1, #2, #3) that compared either sense (S) and antisense (AS), or sense and beads alone **(B)**, resulting in three proteins: ACLY, FLG2, and SFPQ. **(B)** Protein-protein interaction network-based disease associations of three *lnc-FAM164A1*-associated proteins (ACLY, FLG2, and SFPQ). Blank spaces indicate non-significant (empirical p-values > 0.050) associations, and a darker color indicates increasing empirical p-value. “0.00” in the color bar represents emp. p-val < 0.004, which is the detection threshold for N = 250 randomizations. **(C)** Input control of whole cell lysates from LPS-activated human PBMC-derived macrophages and the eluted proteins from the beads control, antisense *lnc-FAM164A1* and sense *lnc-FAM164A1* were detected by Western blot using antibodies against human ACLY and hnRNPA1, respectively. Data shown are representative of three experiments. **(D)** ACLY silencing combined with enforced expression of *lnc-FAM164A1* was performed on human PBMC-derived macrophages, followed by treatment with or without LPS for 2 hours. RNA expression of *lnc-FAM164A1*, *ACLY*, and *CCL2* were assessed by RT-PCR. n = 6–9 samples from three different experiments, *p* < 0.05 by one-way ANOVA.

To address which proteins interact with *lnc-FAM164A1* in LPS-activated THP-1-differentiated macrophages, we performed *lnc-FAM164A1* RNA pulldown assays using a sense versus antisense strategy that in turn was compared to pulldown assays using beads alone. Three proteins (ATP-citrate synthase: ACLY; Filaggrin-2: FLG2; Splicing factor, proline- and glutamine-rich: SFPQ) were commonly enriched between *lnc-FAM164A1* sense RNA versus antisense RNA (S/AS) and sense RNA versus beads controls (S/B) (**Figure 7A**). Furthermore, these three proteins eluted from *lnc-FAM164A1* sense RNA have significantly high normalized abundance compared with antisense RNA and beads controls (**Supplementary Figure 8A**).

### Network analysis suggests potential associations of *lnc-FAM164A1* with human inflammatory diseases

To computationally predict the role of *lnc-FAM164A1* in human diseases, we then examined the disease associations of the interacting proteins ACLY, FLG2, and SFPQ through network analysis of the average shortest network distance between their modules and disease-related proteins. These computational results suggest potential disease relevance with metabolic and inflammatory disorders, such as atherosclerosis, myocardial infarction, obesity, type 2 diabetes and rheumatoid arthritis (**Figure 7B**).

### Silencing *ACLY* decreases the CCL2 and TNF-α expression induced by the enforced expression of *lnc-FAM164A1* on LPS-activated cells

**Figure 8:**
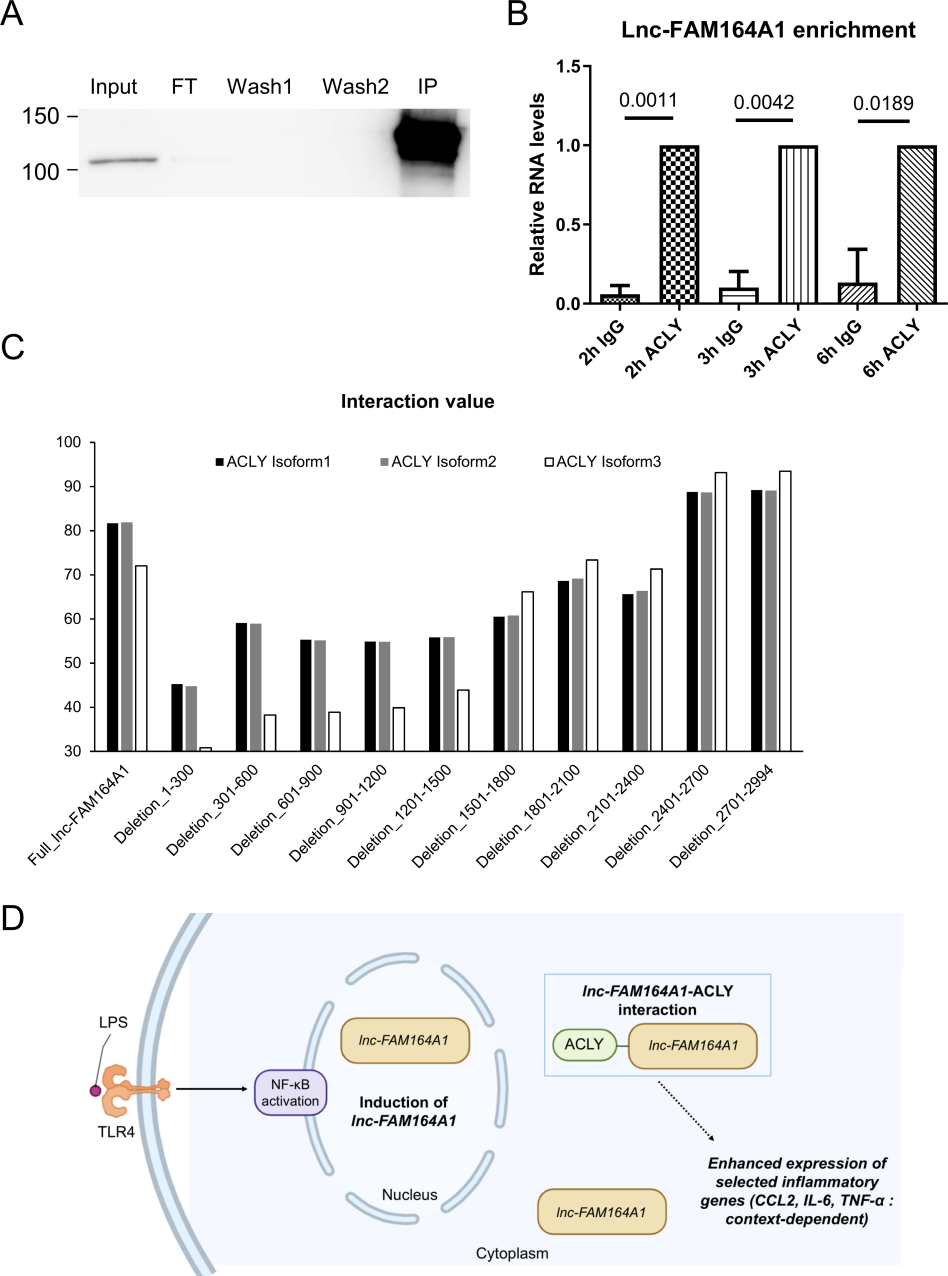
RNA immunoprecipitation (RIP) demonstrates the interaction between ACLY and *lnc-FAM164A1*. **(A)** Western blot of a representative immunoprecipitation of ACLY protein from THP-1 differentiated macrophages stimulated with 10 ng/mL for 3 hours. ACLY signal in input (before immunoprecipitation), flow-through (FT), washes, and IP sample. **(B)** *Lnc-FAM164A1* levels from immunoprecipitations after 2-, 3- and 6-hours stimulation with LPS was measured by qPCR (P<0.05, IgG control vs ACLY, n = 3 independent experiments). **(C)** the sequence of ACLY, full-length *lnc-FAM164A1* and *lnc-FAM164A1* with partial deletion were submitted into lncPro. **(D)** Proposed working model of the *lnc-FAM164A1*–ACLY–NF-κB axis in LPS-activated macrophages. Created in BioRender.com.

RNA pulldown combined with Western blot confirmed the interaction of ACLY with *lnc-FAM164A1* sense RNA in human PBMC-derived macrophages (**Figure 7C**) and THP-1-differentiated macrophages (**Supplementary Figure 8B**). In contrast, heterogeneous nuclear ribonucleoprotein A1 (hnRNPA1) was pulled down by both antisense and sense RNA of *lnc-FAM164A1* (**Figures 7C**, **Supplementary Figure S8B**). Enforced expression of *lnc-FAM164A1* increased ACLY protein expression with no change in *ACLY* mRNA levels in LPS-activated macrophage like cells THP-1 (**Supplementary Figure 10C**). Finally, silencing of *ACLY* significantly decreased the *CCL2* mRNA and TNF-α expression induced by the enforced expression of *lnc-FAM164A1* on human primary macrophages (**Figure 7D**) and THP-1 cells (**Supplementary Figures 8D–F**), respectively. To investigate the interaction between *lnc-FAM164A1* and ACLY, RNA immunoprecipitation (RIP) using anti-ACLY antibody was performed in LPS-stimulated THP-1-differentiated macrophages (**Figure 8A**). As compared to control IgG, *lnc-FAM164A1* was enriched in the IP using anti-ACLY antibody (**Figure 8B**). To investigate their interaction in detail, lncRNA-protein interaction prediction was performed using LncPro (43). The interaction value of *lnc-FAM164A1* with ACLY was higher than other proteins (**Supplementary Figure 9A**). The deletion of 1-300, especially 201–300 nt decreased the interaction value (**Figure 8C**, **Supplementary Figure S9B**).

## Discussion

In the present study, we aimed to identify early responses of lncRNAs as potential key regulators of pro-inflammatory activation of human macrophages. Unbiased screening of LPS-elicited human primary macrophages using human lncRNA microarray identified 11 lncRNAs, including our target lncRNA *lnc-FAM164A1*. Currently, limited information is available for *lnc-FAM164A1* in macrophages. Thus far, the elevated expression of *lnc-FAM164A1* has been reported in Achilles tendons of old human donors and in the macrophage-like THP-1 cells infected with Gram-negative bacterium *Campylobacter concisus (*35, 44). lncRNA-lncRNA network analysis using ovarian cancer datasets from TCGA suggests a potential association of *lnc-FAM164A1* with cell proliferation (45), while a separate study in bladder cancer showed that *lnc-FAM164A1* (*RP11-79H23.3*) suppresses cell proliferation, migration and metastasis *in vivo* by acting as a sponge for *miR-107* and restores *PTEN* expression, which inhibits the PI3K/Akt pathway (37). Liu et al. reported that *IL-7-AS* (alias for *lnc-FAM164A1*) promotes inflammatory gene expression in U937 cells via NF-κB signaling (46). Moreover, in a recent study, *lnc-FAM164A1* (*LINC02605*) promoted IRF3 nuclear translocation under viral infection, leading to enhanced antiviral innate responses. Collectively, these findings suggest that *lnc-FAM164A1* is involved in multiple pathways depending on the cellular or disease contexts (36). In our study, we investigated *lnc-FAM164A1*’s function in human PBMC-derived primary macrophages and THP-1 cells. Loss-of-function (silencing of *lnc-FAM164A1* expression) and gain-of-function (adenovirus-induced enforced expression) experiments in the present study revealed *lnc-FAM164A1* promotes inflammatory responses in LPS-activated human macrophages *in vitro* and mouse models of endotoxemia *in vivo*. ACLY was identified as one of the *lnc-FAM164A1*-associated proteins and *lnc-FAM164A1*/ACLY interaction contributes to the expression of CCL2 and TNF-α induced by enforced expression of *lnc-FAM164A1 in vitro*.

Here we show that *lnc-FAM164A1* is a target for NF-κB signaling and its expression levels increase with LPS, IL-6 or TNF-α stimulation in human primary macrophages. Induced *lnc-FAM164A1* in turn triggers NF-κB signaling cascades supported by several lines of evidence. Our data thus indicates that *lnc-FAM164A1* may enhance inflammation by accelerating a positive feedback loop of NF-κB activation. Multiple lncRNAs (*LOUP* (47), *SUGCT-AS1 (*48), *HIX003209 (*49), *hLinfRNA1 (*22), and *Carlr (*50)) have been demonstrated to regulate NF-κB signaling and the transcription of inflammatory genes in mouse and human macrophages. These findings further support the concept that NF-κB activity is precisely controlled at multiple levels by negative or positive regulatory elements.

Enforced expression of *lnc-FAM164A1* increased the NF-κB activity in unstimulated HEK293 cells and induced the degradation of IκB-α in unstimulated THP-1 cells and primary macrophages (**Figure 4**). However, enforced expression of *lnc-FAM164A1* alone did not increase the expression of *TNF-α* at mRNA and protein levels in cultured macrophages *in vitro* and in mouse plasma samples *in vivo* (**Figure 5**). This data may thus indicate crosstalk between *lnc-FAM164A1* and NF-κB signaling to co-regulate the activation of NF-κB target genes in LPS-stimulated macrophages. Separately, LPS further increased the *lnc-FAM164A1* expression even under adenoviral infection (**Supplementary Figure 5A**). This may reflect both induction of endogenous *lnc-FAM164A1* and LPS responsiveness of the CMV promoter (51). The effects of *lnc-FAM164A1* silencing were not fully identical between gapmer ASOs and siRNAs. One possible explanation is that these two approaches act through different mechanisms and may reduce different pools of the RNA. Since *lnc-FAM164A1* is present in both the nucleus and the cytoplasm, its functional effects may vary depending on which RNA fraction is more strongly affected. Together with the variable responses observed across experimental systems, these findings suggest that *lnc-FAM164A1* may modulate selected NF-κB target genes in a context-dependent manner rather than causing a uniform increase in all inflammatory genes. Another limitation of this study is that we mainly examined CCL2, IL-6, and TNF-α as representative inflammatory readouts. Broader cytokine profiling will be useful in future studies to determine more clearly whether *lnc-FAM164A1* affects a wide inflammatory response or only selected target genes.

We demonstrated that ACLY, as *lnc-FAM164A1*-associated protein, contributes to the expression of CCL2 and TNF-α induced by enforced expression of *lnc-FAM164A1 in vitro* (**Figure 7**, **Supplementary Figure 8**). We also found that enforced expression of *lnc-FAM164A1* increases the protein expression of ACLY without change of the *ACLY* mRNA in THP-1-differentiated macrophage cells (**Supplementary Figure 10**). Although ACLY interacts with *lnc-FAM164A1*, our current data did not define the downstream mechanism. We did not measure ACLY enzymatic activity, acetyl-CoA levels, histone acetylation, or other metabolic outputs in this study. Therefore, we present the *lnc-FAM164A1*–ACLY link as a working model with two possible mechanisms: i) a catalytic role of ACLY, where acetyl-CoA production may support acetylation-dependent regulation of NF-κB–responsive genes; and ii) a post-transcriptional role, where *lnc-FAM164A1* may affect ACLY protein stability and/or translation. We did not determine whether the increase in ACLY protein reflects enhanced translation or increased protein stability; assays such as cycloheximide chase or polysome profiling will be required in future studies. In LPS-activated macrophages, the elevated expression of the citrate carrier (SLC25A1) leads to increased transport of citrate out of the mitochondria (52). Cytosolic citrate can be metabolized by ACLY to generate oxaloacetate and acetyl-CoA. This acetyl-CoA serves as a substrate for histone acetylation, which epigenetically primes pro-inflammatory gene loci (53, 54). Therefore, enforced expression of *lnc-FAM164A1* could influence cytosolic acetyl-CoA level and the acetylation of p65/p50, which promotes NF-κB activation (55) and leads to the enhanced expression of proinflammatory cytokines in LPS-activated human macrophages. However, these downstream acetylation-related mechanisms remain speculative in our study and should be directly tested in future experiments.

A previous study on bladder cancer has demonstrated that PTEN, a negative regulator of PI3K/Akt signaling pathway, and *lnc-FAM164A1* (*RP11-79H23.3*) both contain conserved target sites of *miR-107 (*37). Thus, it is possible that the enforced expression *of lnc-FAM164A1* in LPS-activated monocytes/macrophages could have led to increased *PTEN* expression and inactivation of PI3K/AKT signaling pathway, that in turn would have negatively regulated NF-κB activation (42, 56). Thus, *lnc-FAM164A1* may also act as a sponge for *miR-107* to regulate NF-κB signaling in human macrophages.

Mouse *IL-7-AS* was reported as a syntenic lncRNA of human *IL-7-AS*, however, the majority of the human lncRNAs, including *lnc-FAM164A1*, show only limited alignment and does not support robust sequence-level conservation, which brings challenges to determine the biological significance of lncRNAs in mouse models (57, 58). To this end, we established a humanized mouse model of endotoxemia to determine the *in vivo* significance of *lnc-FAM164A1*. Humanized NSG mice engrafted with CD34+ HSCs or PBMCs have been widely used to study not only human biological processes *in vivo* but also human diseases including infectious diseases, autoimmunity, cancer and atherosclerosis (40, 59–61). In a humanized NSG mouse model of endotoxemia (61), LPS challenge increased the expression of *lnc-FAM164A1* in blood leukocytes (**Figure 1D**). The expression levels of *lnc-FAM164A1* were higher in spleens than in lungs (**Supplementary Figure 2D**) of endotoxemic NSG mice, which is consistent with the known role of the spleen as a major reservoir of human monocyte/macrophages that can be activated and mobilized during systemic inflammation (62). We further demonstrated that siRNA silencing of *lnc-FAM164A1* by lipid nanoparticles specifically targeting monocyte/macrophages can result in a significant decrease of human CCL2 and IL-6 expression in plasma and peritoneal cells isolated from endotoxemic NSG-SGM3 mice (**Figure 6**). Further analysis of human cytokine mRNA in different organs have found that silencing of *lnc-FAM164A1* decreases the mRNA of proinflammatory cytokines in the lungs, but without change in liver and spleen (**Supplementary Figure 11**) suggesting alveolar macrophages significantly contribute to the systematic inflammation and septic lung injury. We also found increased expression of *lnc-FAM164A1* in PBMCs isolated from LPS-stimulated citrated human whole blood *ex vivo* (**Figure 1C**). The data suggests that the expression of *lnc-FAM164A1* increases in the circulating PBMCs from septic patients. Although *lnc-FAM164A1* expression levels in blood could potentially be explored as a biomarker for systemic inflammation, this premise is speculative and warrants further clinical investigations. Our *in vivo* experiments were limited to endotoxemia-related models, and disease-specific validation will be required to test broader disease relevance.

Other previous studies have focused on the association of lncRNAs and RNA-binding proteins (RBPs) and their mechanisms (63, 64). The evidence for the mechanism via the interaction with RBPs remains limited to only a small number of lncRNAs. In this study, we demonstrated that ACLY could be associated with *lnc-FAM164A1* (**Figure 8**). As Zheng et al. reported that ACLY interacts with lncRNA *TINCR* and regulates tumorigenesis, ACLY may have the potential to bind to RNA and regulate bioactivities (65). Our results of the LncPro prediction indicated that *lnc-FAM164A1* and ACLY may interact directly, but this interaction requires the 1–300 nucleotide site of *lnc-FAM164A1* (**Figure 8C**). Understanding the interaction between *lnc-FAM164A1* and ACLY requires additional examinations.

We acknowledge certain limitations in our current study. While we established that *lnc-FAM164A1* regulates the transcription and secretion of key pro-inflammatory cytokines, which is a critical functional output of macrophages in endotoxemia models, we did not perform broader functional assays such as phagocytosis and chemotaxis. Incorporating these functional assays will be an important direction for future investigations to fully understand the biological relevance and comprehensive mechanisms of *lnc-FAM164A1* during inflammation. Although we reported dual nuclear/cytoplasmic localization of *lnc-FAM164A1*, we did not dissect compartment-specific functions. Another limitation of this study is the small size of the initial screening cohort, which included only four donors. Therefore, our findings should be validated in larger independent cohorts.

Collectively, our results support a role for human lncRNA *lnc-FAM164A1* in modulating proinflammatory gene expression in human primary macrophages through its interaction with ACLY and NF-κB -elated signaling (**Figure 8D**). Our study thus suggests a potential molecular basis for the development of *lnc-FAM164A1*-targeted therapeutics for inflammatory diseases mediated by activated macrophages.

## Data Availability

The microarray data presented in this study are publicly available in the GEO under accession number GSE328798.
