# Supplementary figures and images for: The long noncoding RNA lnc-FAM164A1-ACLY axis promotes pro-inflammatory responses in human primary macrophages: a systems approach
Source: Front Immunol. 2026 May 1;17:1776849. doi: 10.3389/fimmu.2026.1776849 (PMC13175867; doi:10.3389/fimmu.2026.1776849)

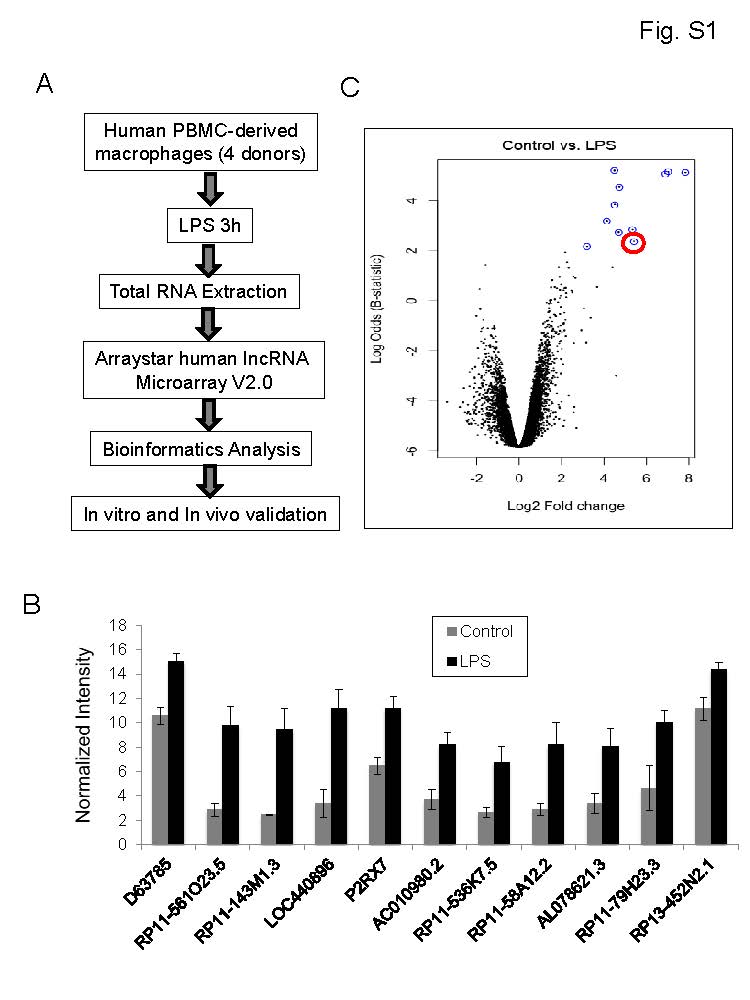

Supplement: Supplementary file 1 [file Image1.jpeg]

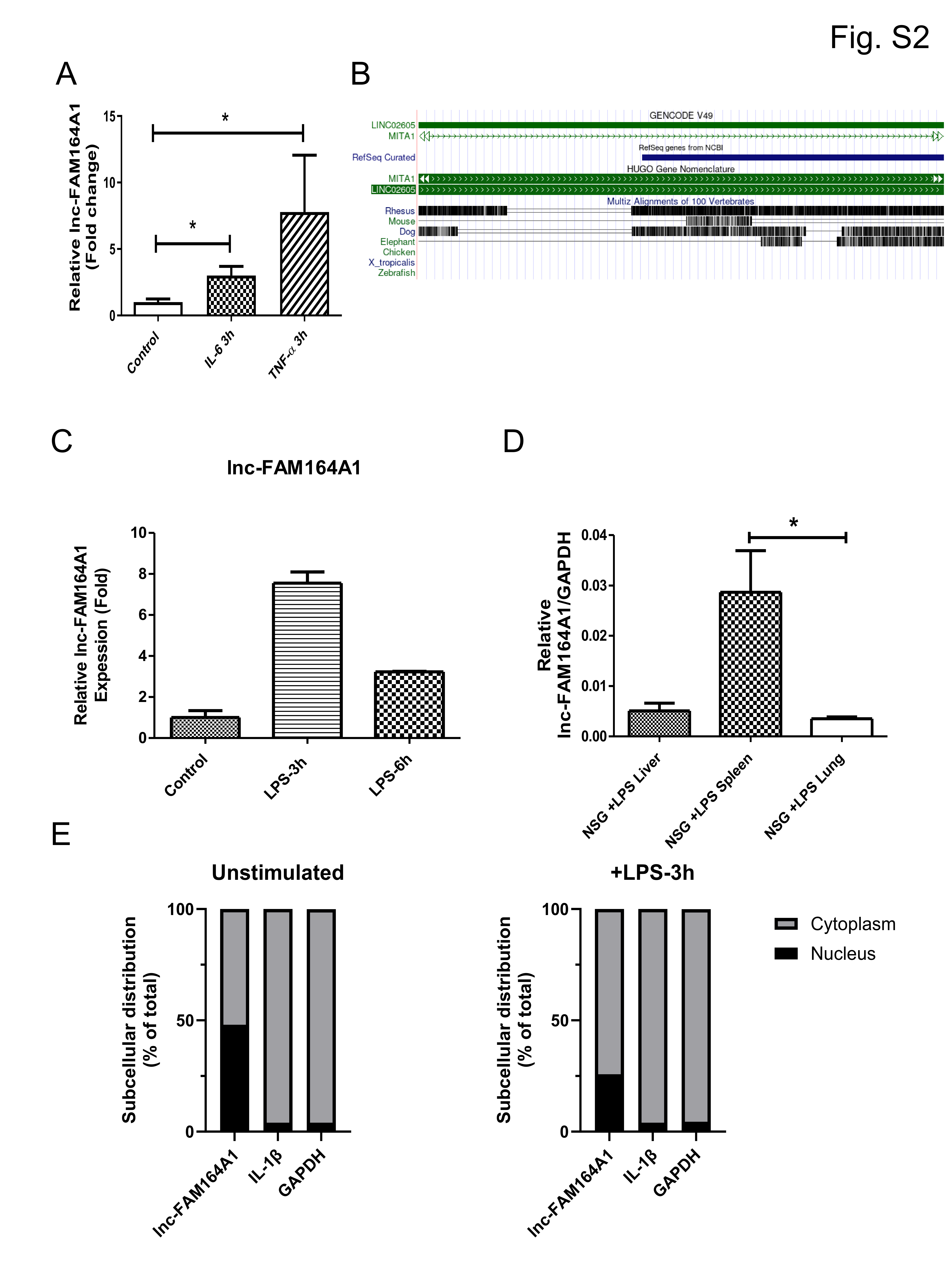

Supplement: Supplementary file 2 [file Image2.tiff]

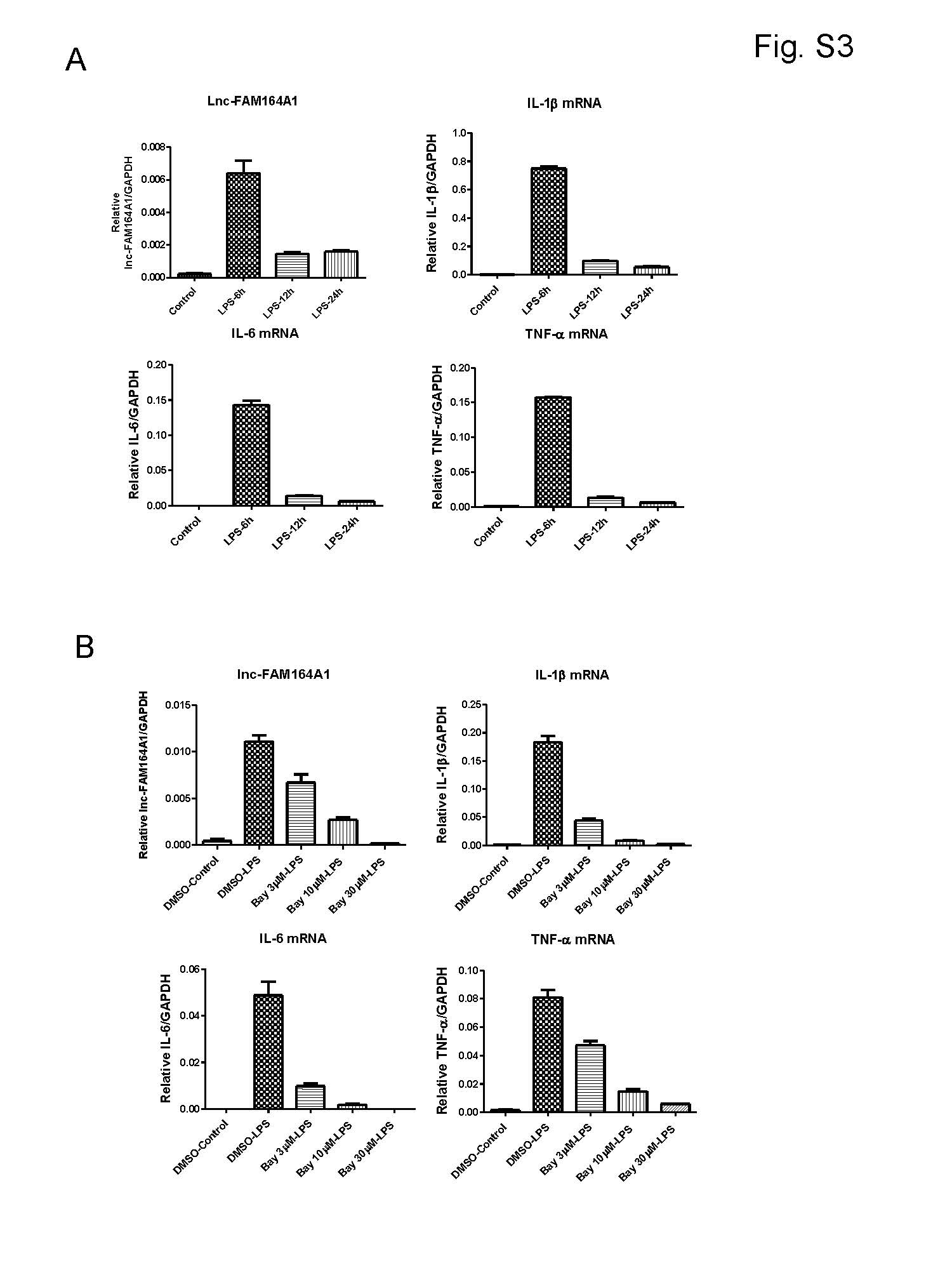

Supplement: Supplementary file 3 [file Image3.jpeg]

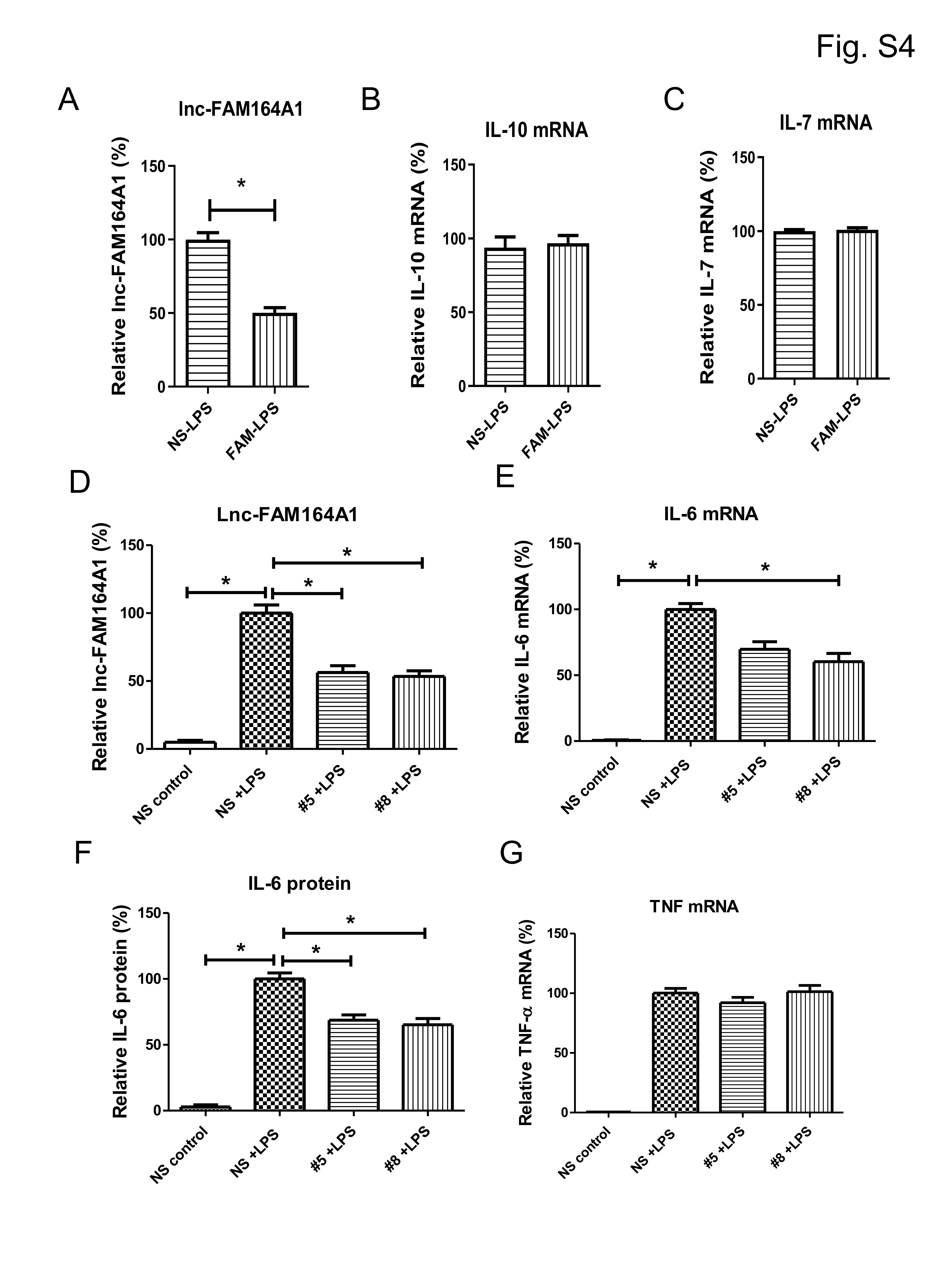

Supplement: Supplementary file 4 [file Image4.tiff]

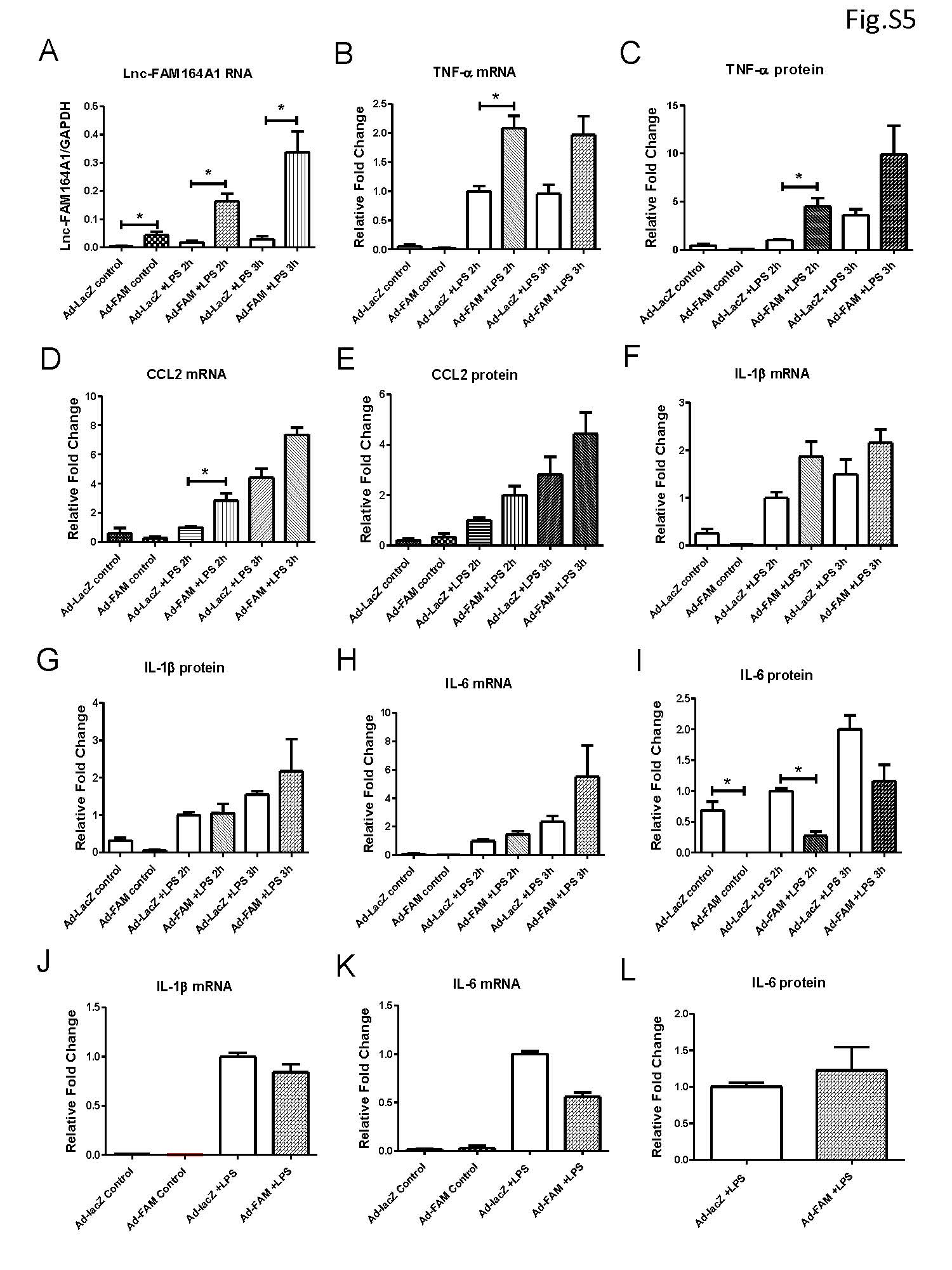

Supplement: Supplementary file 5 [file Image5.jpeg]

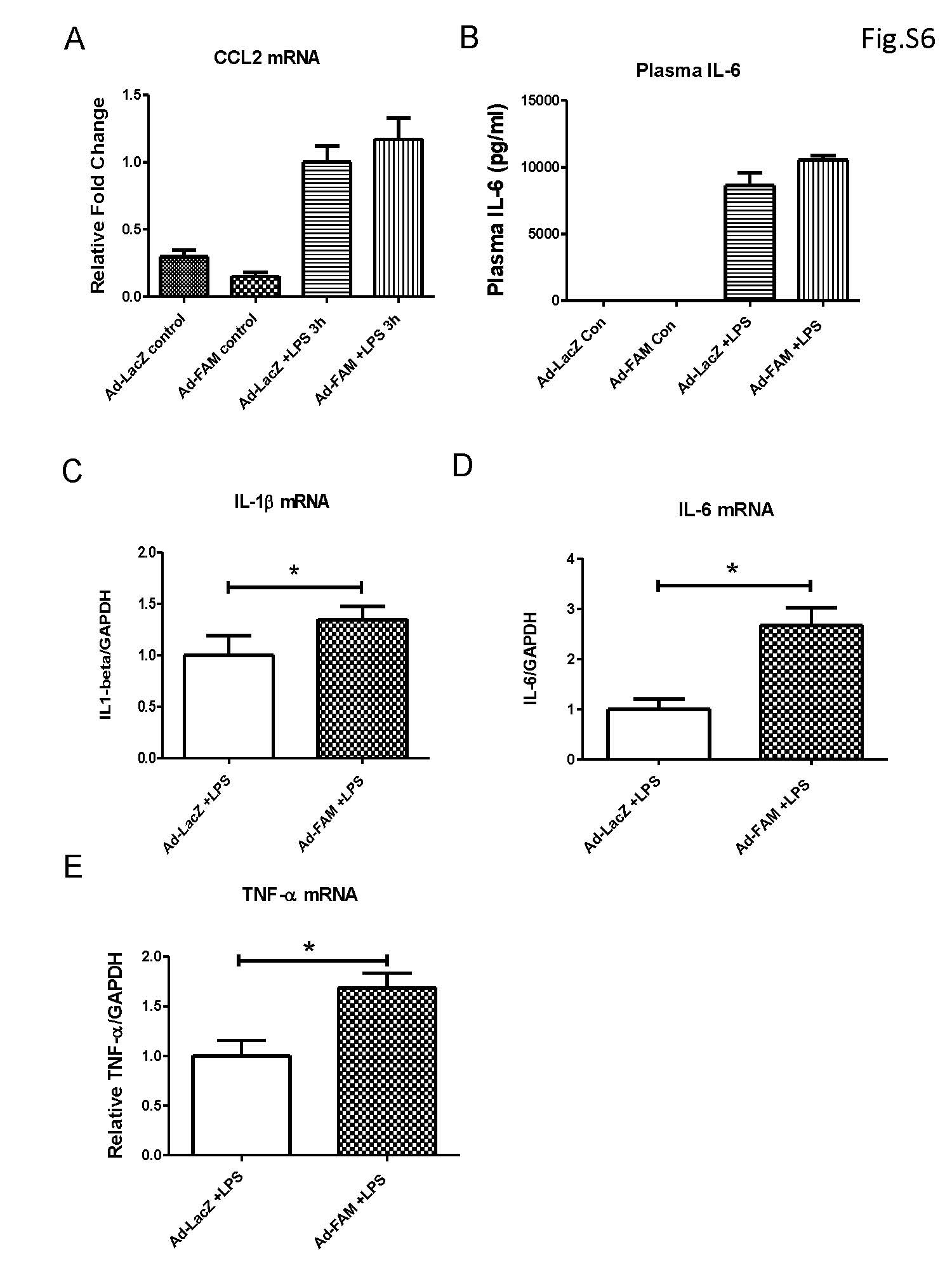

Supplement: Supplementary file 6 [file Image6.jpeg]

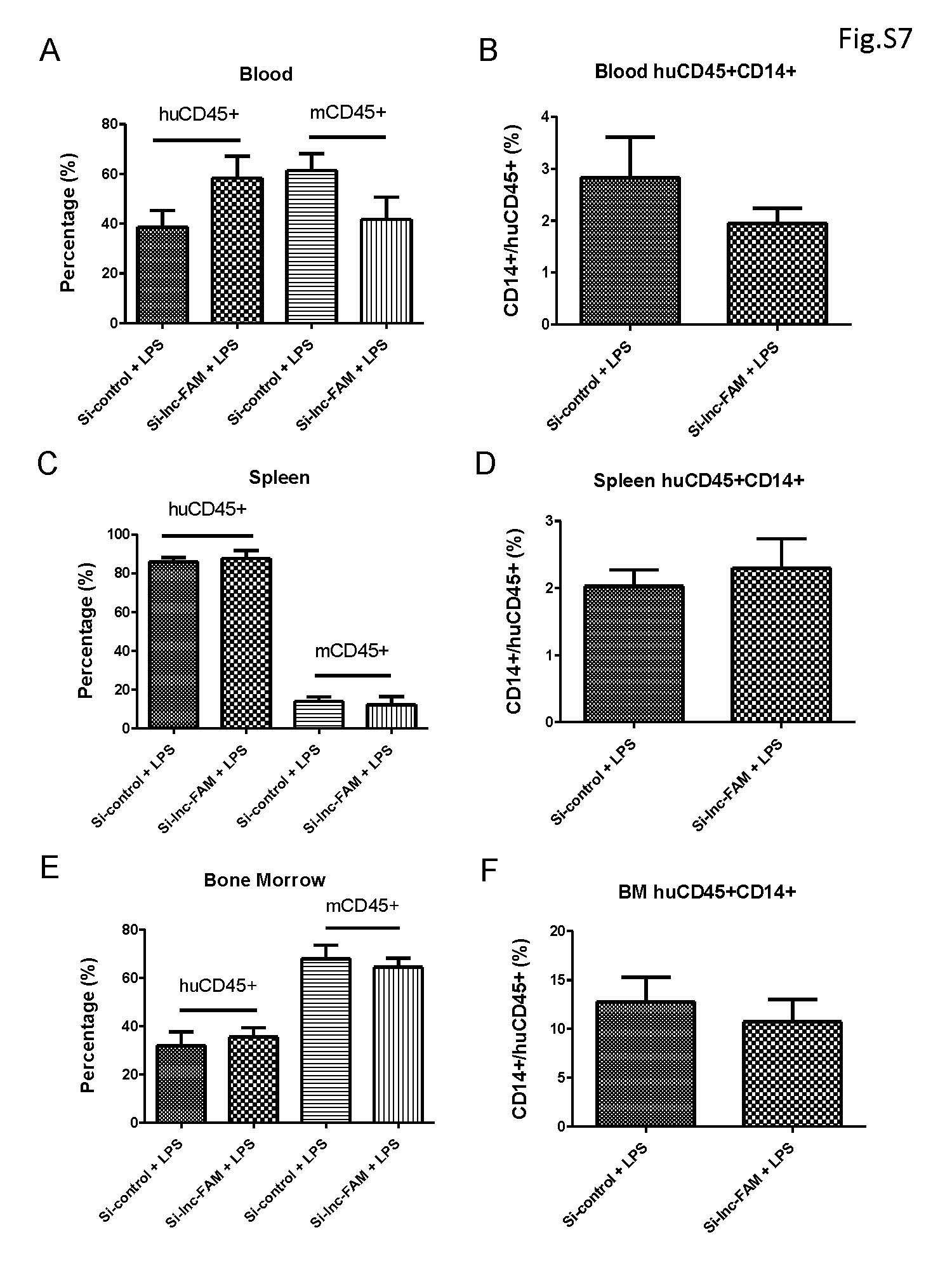

Supplement: Supplementary file 7 [file Image7.jpeg]

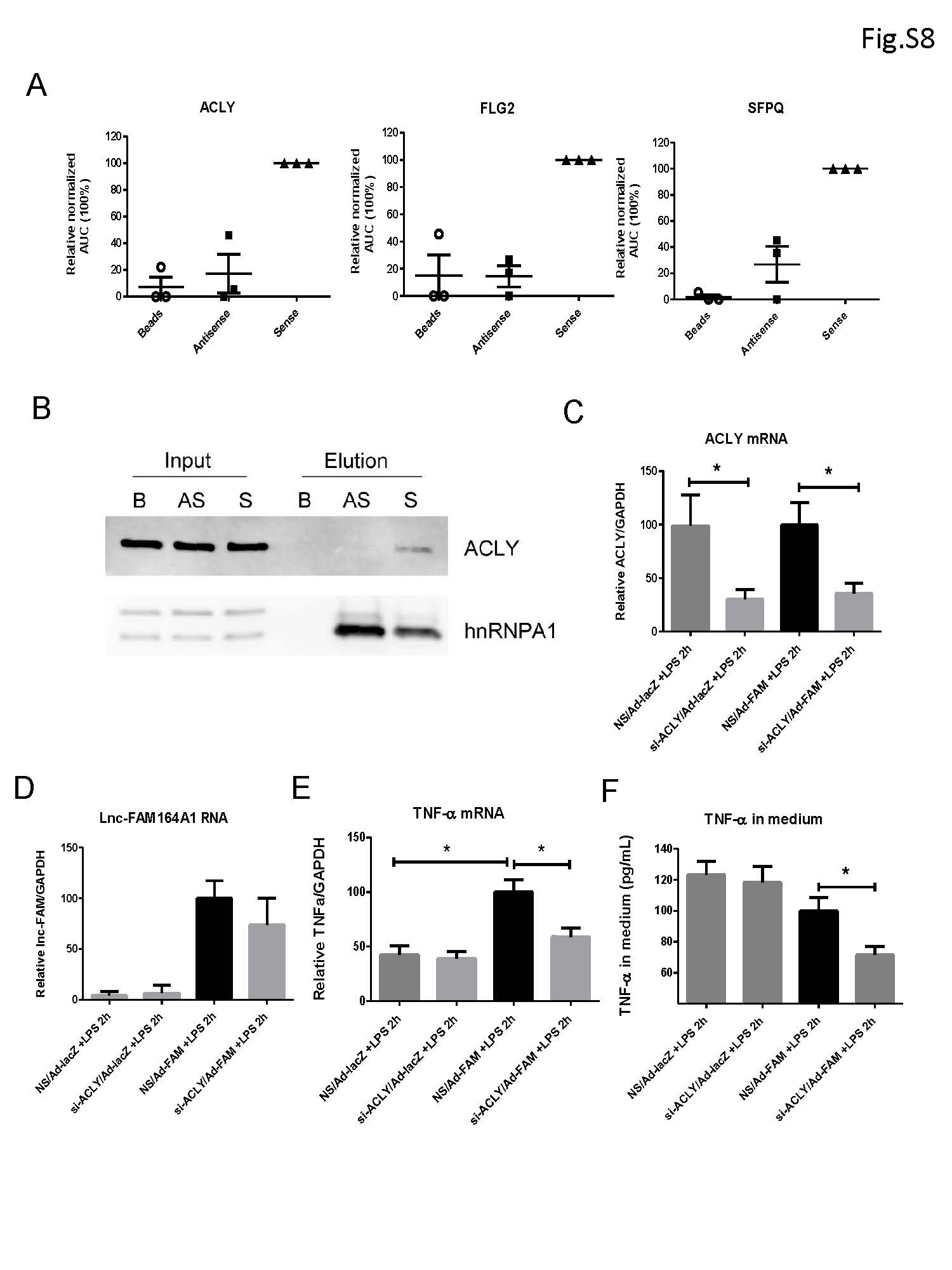

Supplement: Supplementary file 8 [file Image8.jpeg]

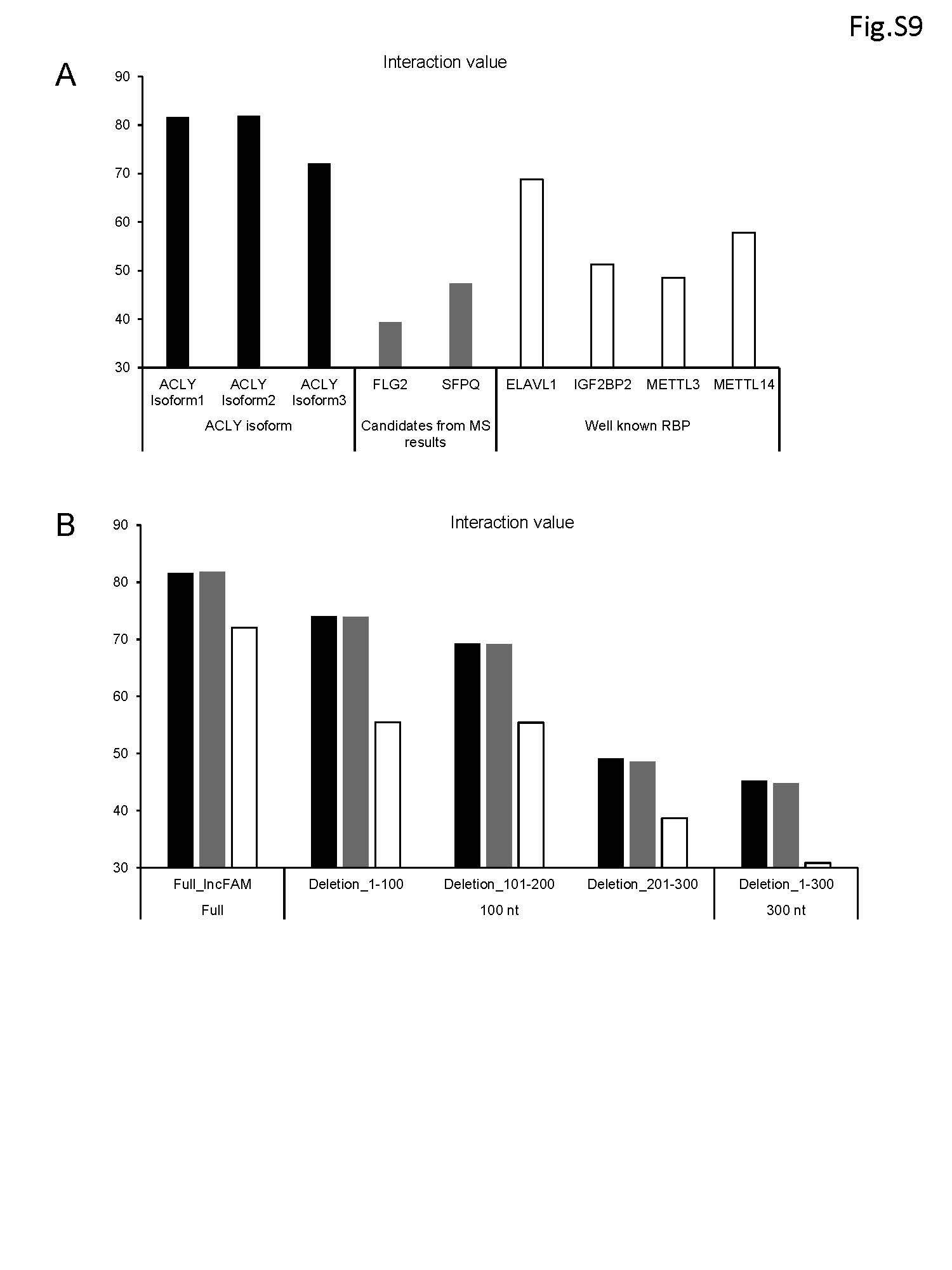

Supplement: Supplementary file 9 [file Image9.jpeg]

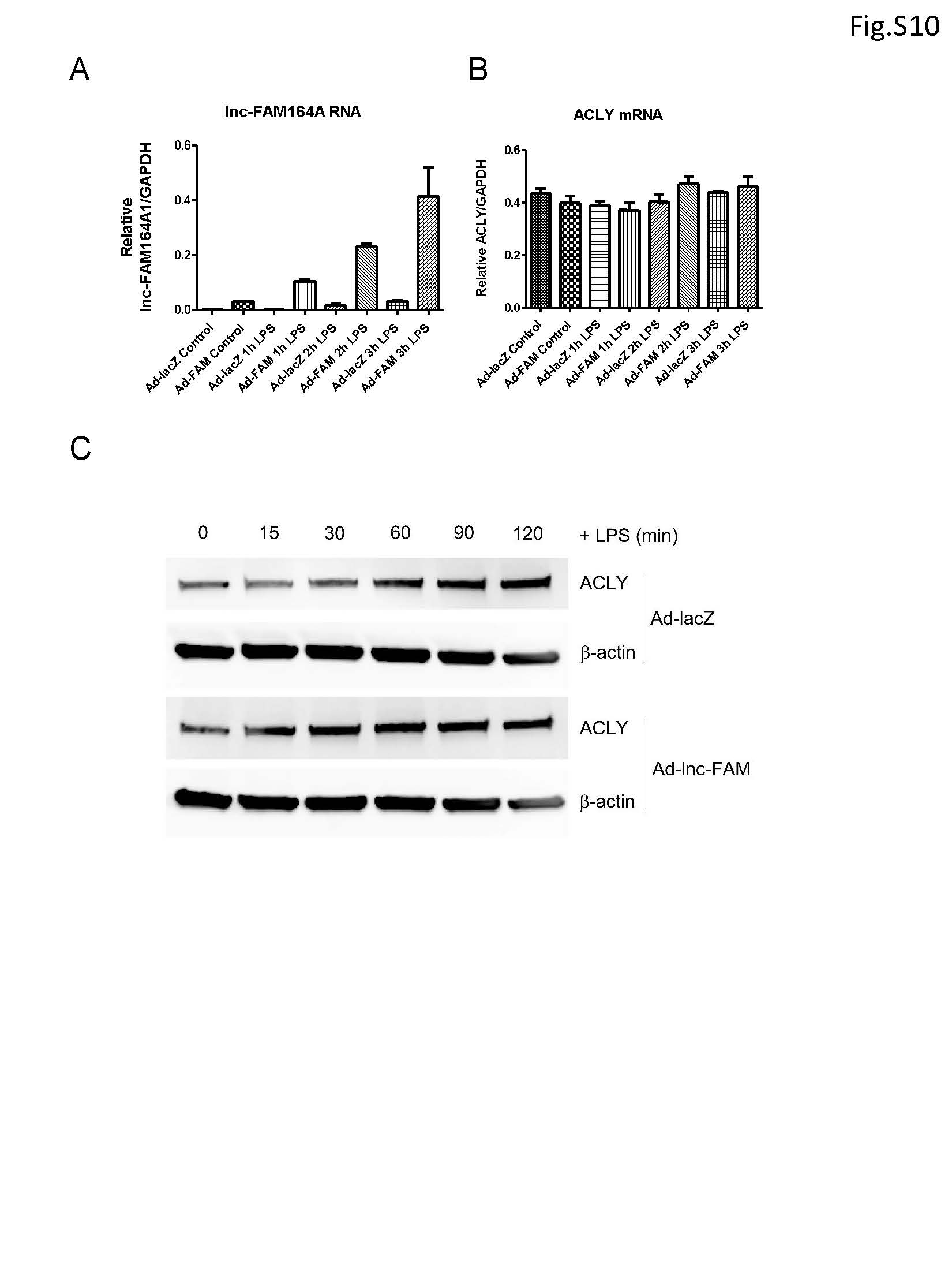

Supplement: Supplementary file 10 [file Image10.jpeg]

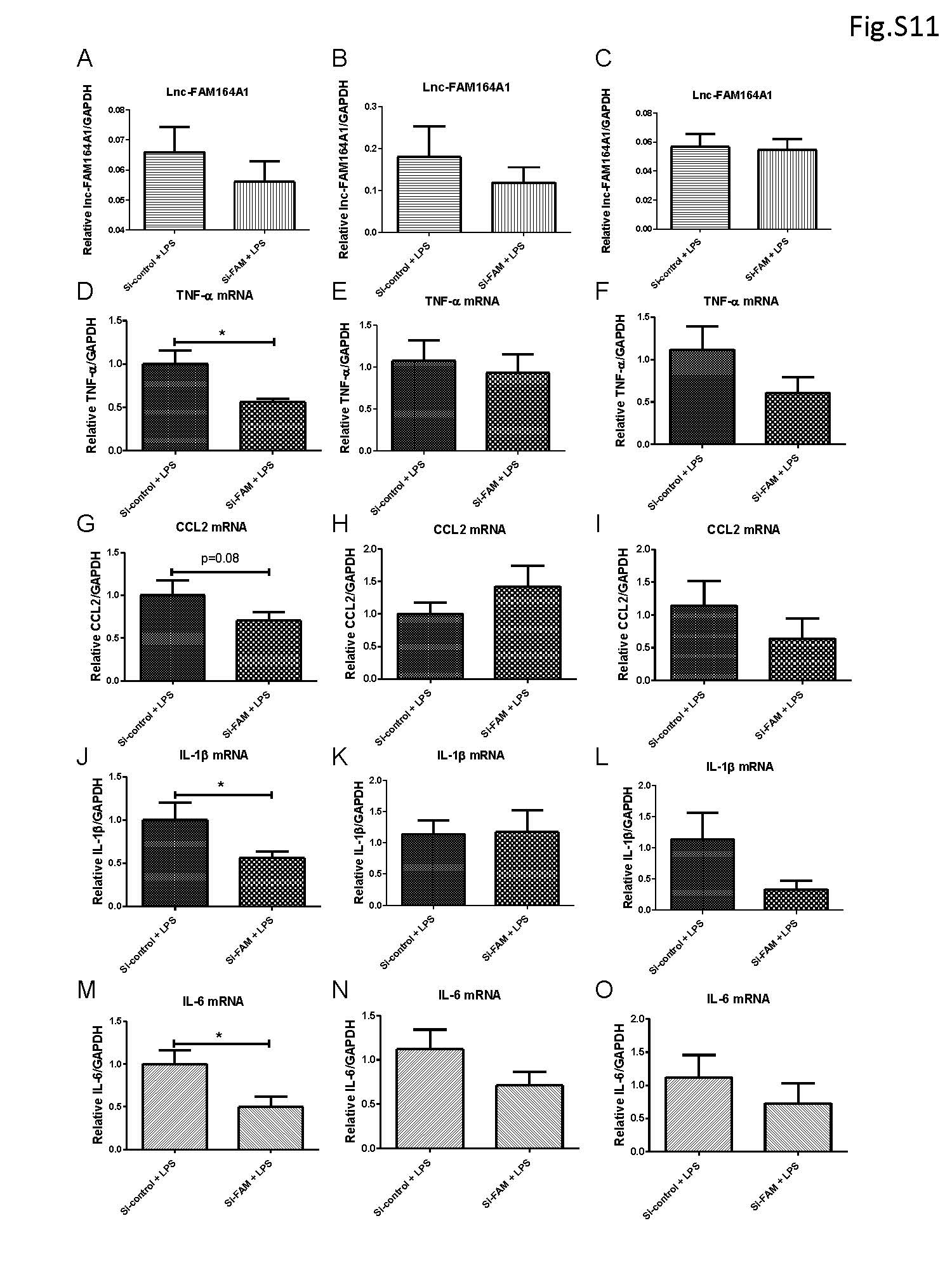

Supplement: Supplementary file 11 [file Image11.jpeg]
