# Supplementary material for: The long noncoding RNA lnc-FAM164A1-ACLY axis promotes pro-inflammatory responses in human primary macrophages: a systems approach
Source: Front Immunol. 2026 May 1;17:1776849. doi: 10.3389/fimmu.2026.1776849 (PMC13175867; doi:10.3389/fimmu.2026.1776849)
Supplement: Supplementary file 12 [file Table1.docx]

Supplementary Material

# Materials and Methods

**Global microarray screening**

mRNA was purified from total RNA after removal of rRNA (mRNA-ONLY™ Eukaryotic mRNA Isolation Kit, Epicentre). Then, each sample was amplified and transcribed into fluorescent cRNA along the entire length of the transcripts without 3’ bias utilizing a random priming method. The labeled cRNAs were hybridized onto the Human LncRNA Array v3.0 (8 x 60K, Arraystar). After having washed the slides, the arrays were scanned by the Agilent Scanner G2505C. Agilent Feature Extraction software (version 11.0.1.1) was used to analyze acquired array images. Quantile normalization and subsequent data processing were performed using the GeneSpring GX v11.5.1 software package (Agilent Technologies). After quantile normalization of the raw data, LncRNAs and mRNAs that at least 4 out of 8 samples have flags in Present or Marginal (“All Targets Value”) were chosen for further data analysis. We employed “limma” R package to analyze the microarray data. We followed the steps of the manual and chose significant 11 RNAs that have lower adjusted p-value than 0.05 and higher log2 fold change than 1. We drew volcano plot and QQ plot using functions provided by limma package.

# Supplemental Figures

**Figure S1**: *A,* **Experimental scheme**. *B,* Normalized intensity of each lncRNA in human lncRNA microarray analysis was shown as mean ± SD (p<0.05, LPS *vs* control n= 4 different donors). *C,* Volcano plot of lncRNAs based on their relative expression levels (Log2 fold-change, LPS vs Control). 11 lncRNAs (Blue circles) have significant increase of expression induced by LPS treatment in human PBMC-derived macrophages (n=4, adjusted p-value FDR < 0.05 and Fold-change ≥ 2). Lnc-FAM164A1 highlighted in Red.

**Figure S2:** *A,* Human PBMC-derived macrophages were stimulated with 10 ng/mL human IL-6 and TNF-α for 3 hours. Relative fold change of the lnc-FAM164A1 expression was determined by RT-PCR (*P*<0.05 LPS *vs* control; n= 3-4 different donors). *B,* Rhesus Monkey PBMC-derived macrophages were stimulated with 10 ng/mL LPS for 3 and 6 hours. Relative fold change of the lnc-FAM164A1 expression was determined by RT-PCR. *C,* Hu-CD34 NSG mice were stimulated with LPS for 6 hours. Expression of lnc-FAM164A1 RNA in liver, spleen and lung isolated from the endotoxemic mice was determined by RT-PCR (*P*<0.05, Spleen vs Lung; n = 4 mice per group). *D,* Ratio of lnc-FAM164A1 in cytoplasm and nucleus of PBMC-derived macrophages. Human PBMC-derived macrophages were stimulated with 10 ng/mL LPS for 3 hours. Nuclear and cytoplasmic RNA were separately purified. Lnc-FAM164A1 RNA, *IL-1β*, and *GAPDH* mRNA expression were determined by RT-PCR and shown as the ratio of nuclear/cytoplasmic in unstimulated macrophages (Left Panel) and LPS-treated macrophages (Right Panel) (n= 2 donors).

**Figure S3: Time course of lnc-FAM164A1 and cytokines expression in LPS-stimulated PBMC-derived macrophages.** *A,* Human PBMC-derived macrophages were stimulated with 10 ng/mL LPS for 6, 12 and 24 hours. Lnc-FAM164A1 RNA, *IL-1β*, *IL-6*, and *TNF-α* mRNA expression were determined by RT-PCR and normalized by *GAPDH* expression. (n = 3 technical replicates). *B,* **NF-κB inhibitor (**Bay 11-7802) blocks the transcription of lnc-FAM164A1 and cytokines in LPS-stimulated PBMC-derived macrophages. Human PBMC-derived macrophages were pretreated with 3-30 μM Bay 11-7802 for 30 minutes before the stimulation with 10 ng/mL LPS for 6 hours. Lnc-FAM164A1 RNA, *IL-1β*, *IL-6*, and *TNF-α* mRNA expression were determined by RT-PCR and normalized by *GAPDH* expression (n = 3 technical replicates).

**Figure S4: Antisense or siRNA oligonucleotides silencing of lnc-FAM164A1 reduces the expression of CCL2, IL-6 and TNF-α on human PBMC-derived macrophages.**

*A and B,* Human PBMC-derived macrophages were transfected with lnc-FAM164A1 antisense oligonucleotides (FAM).*C-F*, Axolab siRNA oligonucleotides (#5 and #8) or their nonspecific control oligonucleotides (NS) for 48h followed by 3-6 hours stimulation with LPS. Levels of lnc-FAM164A1 RNA (*A and C*), *IL-10* mRNA (*B*), *IL-6* mRNA (*D*), and *TNF-α* mRNA (*F*) were detected by RT-PCR (*P*<0.05 NS-LPS *vs* FAM-LPS; n = 5-6 different donors). IL-6 protein (*E*) in culture medium were measured by ELISA (*P*<0.05 NS-LPS *vs* #5+LPS or #8+LPS; n = 4 donors).

**Figure S5: Effect of enforced expression of lnc-FAM164A1 on TNF-α, CCL2, IL-1β and IL-6 expression in THP-1-derived macrophages and human PBMC-derived macrophages.** *A-I,* RNA and protein expressions in human monocytic THP-1-derived macrophages. *J-L,* RNA, and protein expressions in human PBMC-derived macrophages. The cells were infected with LacZ control adenovirus (Ad-lacZ) or lnc-FAM164A1 expressing adenovirus (Ad-lnc-FAM164A1) for 48 hours and then stimulated with 10 ng/mL LPS for 2-3 hours. Expression levels of lnc-FAM164A1 RNA (*A*) and *TNF-α*, *CCL2*, *IL-1β*, and *IL-6* mRNA (*B, D, F, H, J and K*) were determined by RT-PCR. Protein levels of TNF-a, CCL2, IL-1β and IL-6 in culture medium were measured by ELISA (*C, E, G, I and L*). (*P*<0.05 Ad-LacZ *vs* Ad-lnc-FAM164A1; n = 5-7 different experiments).

**Figure S6: Effect of enforced expression of lnc-FAM164A1 on mouse peritoneal macrophages***. A,* Expression levels of CCL2 mRNA in peritoneal macrophages infected with LacZ control adenovirus (Ad-lacZ) or lnc-FAM164A1 expressing adenovirus (Ad-*lnc-FAM164A1*) for 48 hours and then stimulated with 10 ng/mL LPS for 3hours. *B*, Plasma levels of IL-6 (measured by ELISA) from C57BL/6J mice were infected with LacZ control adenovirus (Ad-lacZ) or lnc-FAM164A1 expressing adenovirus (Ad-lnc-FAM164A1) for 3 days and then stimulated with LPS for 3 hours. *C-E*, Corresponding mouse liver mRNA of *IL-1β*, *IL-6*, and *TNF-α* mRNA in liver were determined by RT-PCR. (*P*<0.05 Ad-LacZ *vs* Ad-lnc-FAM164A1; n = 9-10 mice per group).

**Figure S7: Numeration of Human CD45+ WBC and monocytes in humanized Hu-CD34 NSG-SGM3 mice.** Hu-CD34 NSG-SGM3 mice were administrated with lipid nanoparticle formulated siRNA control oligos or si-lnc-FAM oligos, followed by LPS challenge for 3 hours. The percentage of human CD45+ and mouse CD45+ WBCs (*A, C and E*) and human CD45+CD14+ monocytes (*B, D and F*) in the blood, spleen and bone marrow were detected by Flow Cytometry. (*P*<0.05, si-control+LPS *vs* si-lnc-FAM +LPS, n = 6-7 mice per group).

**Figure S8: Mass spectrometry (MS) identified three proteins associated with lnc-FAM164A1.** *A*, Relative normalized abundance of ACLY, FLG2 and SFPQ in Beads control (Beads), Antisense lnc-FAM164A1 (Antisense) and sense lnc-FAM164A1 (Sense) detected by MS (n=3, *p*<0.05 by *t*-test, Sense vs Beads; Sense vs Antisense). AUC, area-under-the-curve of the extracted MS1 ion peak chromatograms of up to the top-3 peptides of each protein. *B*, Input control of whole cell lysates from LPS-activated THP-1-differentiated macrophages and the eluted proteins from Beads control (Beads), Antisense lnc-FAM164A1 (Antisense) and sense lnc-FAM164A1 (Sense) were detected by Western blot using antibodies against human ACLY and hnRNPA1, respectively. The data shown was representative of three experiments. *C-F*, ACLY silencing combined with enforced expression of lnc-FAM164A1 was performed on THP-1-differentiated macrophages, followed by treatment with or without LPS for 2 hours. RNA expression of *ACLY*, lnc-FAM164A1, and *TNF-α* were assessed by RT-PCR. TNF-α protein in culture medium was measured by ELISA (n = 8-9 samples from three different experiments, *p*<0.05 by one-way ANOVA).

**Figure S9: Prediction using LncPro demonstrated the interaction of lnc-FAM164A1** **and ACLY.** *A,* the sequence of lnc-FAM164A1, ACLY, 3 candidate proteins in Figure 7B and well-known RNA binding proteins were submitted into LncPro. *B,* the sequence of ACLY, full-length lnc-FAM164A1 and lnc-FAM164A1 with partial deletion were submitted into lncPro.

**Figure S10: Enforced expression of lnc-FAM164A1 increases the protein expression of ACLY without change of the ACLY mRNA.** *A and B,* THP-1-derived macrophages were infected with LacZ control adenovirus (Ad-lacZ) or lnc-FAM164A1 expressing adenovirus (Ad-lnc-FAM164A1) for 48h and then stimulated with 10 ng/mL LPS for 1-3 hours. Expression levels of lnc-FAM164A1 RNA (*A*) and ACLY (*B*) were determined by RT-PCR. *C,* Protein levels of ACLY were analyzed by western blotting.

**Figure S11: Silencing of lnc-FAM164A1 decreases mRNA of proinflammatory cytokines in the lungs, but without change in liver and spleen in a humanized mouse model of endotoxemia.** *A-C,* Expression level of lnc-FAM164A1; *D-F, TNF-α*; *G-I*, *CCL2*; *J-L,* *IL-1β* ; and *M-O, IL-6*. Hu-CD34 NSG-SGM3 mice were administrated with lipid nanoparticle formulated siRNA control oligos or si-lnc-FAM oligos, followed by LPS challenge for 3 hours. RNA levels in lung (*A, D, G, J, M*), liver (*B, E, H, K, N*) and spleen (*C, F, I, L, O*) were determined by RT-PCR. (*P*<0.05, si-control +LPS *vs* si-*lnc-FAM* +LPS, n = 6-7 mice per group).
